# Exceptionally well-preserved crocodilian coprolites from the Late Eocene of Northern Vietnam: Ichnology and paleoecological significance

**DOI:** 10.1016/j.isci.2023.107607

**Published:** 2023-08-11

**Authors:** Kazım Halaçlar, Paul Rummy, Jia Liu, Adrian P. Hunt, Truong Van Do, Nguyen Trung Minh, Tao Deng

**Affiliations:** 1 Key Laboratory of Vertebrate Evolution and Human Origins, Institute of Vertebrate Paleontology and Paleoanthropology, Chinese Academy of Sciences, Beijing 100044, People’s Republic of China; 2 College of Earth and Planetary Sciences, University of Chinese Academy of Sciences, Beijing 100049, People’s Republic of China; 3 CAS Key Laboratory of Tropical Forest Ecology, Xishuangbanna Tropical Botanical Garden, Chinese Academy of Sciences, Mengla 666303, People’s Republic of China; 4 Flying Heritage and Combat Armor Museum, Everett, WA 98204, USA; 5 Vietnam National Museum of Nature, Vietnam Academy of Science and Technology, Hanoi 113000, Vietnam; 6 Graduate Academy of Science and Technology, Vietnam Academy of Science and Technology, Hanoi 113000, Vietnam

**Keywords:** Natural sciences, Biological sciences, Paleobiology

## Abstract

This study examines 55 coprolites from the Na Duong Basin to reconstruct the paleoenvironment. Coproecology sheds light on understanding the complex prey-predator relationships, trophic dynamics, and ecosystem evolution. Through quantitative and multidisciplinary analysis, the putative coprolites were attributed to crocodilian producers, leading to the establishment of a new ichnogenus and species, *Crococopros naduongensis* igen. et isp. nov., based on distinct characteristics and comparisons. The study provides compelling evidence of an ancient river or lake-like environment dominated by diverse crocodilian fauna, indicating a thriving food chain in the Na Duong Basin. The findings also highlight the remarkable richness of ichnofauna, fauna, flora, and the presence of a favorable climate, confirming the area as a significant fossil Lagerstätte in Southeast Asia. Overall, this study offers a unique snapshot of the past, providing valuable insights into the regional ecosystem and significantly contributing to our understanding of paleoenvironmental conditions and biotic interactions.

## Highlights

• New ichnogenus and ichnospecies *Crococopros naduongensis*
• First quantitative study on crocodilian coprolites
• Multidisciplinary approach to understand paleoecology of Na Duong Basin
• Tangible evidence supporting Na Duong Basin as a fossil-Lagerstätte of Southeast Asia

## Graphical abstract

**Figure:**
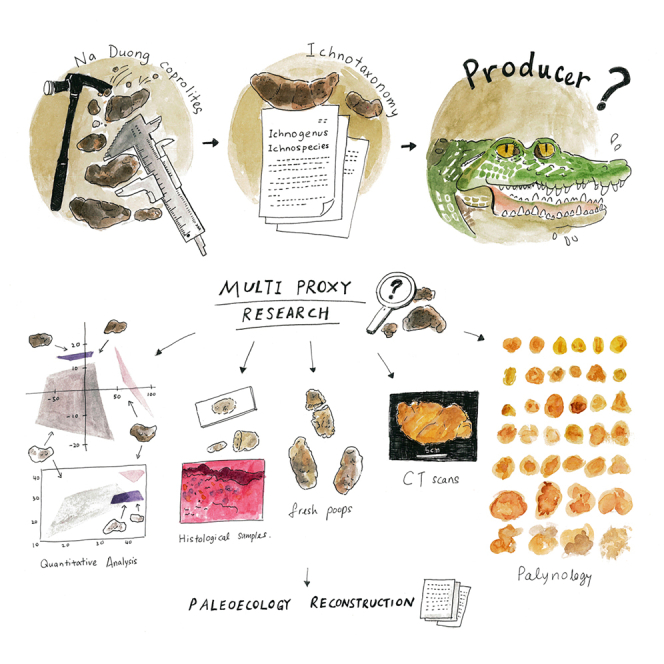

## Introduction

Vertebrate coprolites, or fossilized feces, have a long history of study spanning several centuries.^1^^,^^2^^,^^3^^,^^4^ However, it has often erroneously been considered that feces have low preservation potential.^5^ Recent advancements have led to their extensive utilization in multidisciplinary research programs.^6^^,^^7^^,^^8^ These coprolites have proven to be invaluable tools in paleobiological studies,^9^^,^^10^^,^^11^^,^^12^ offering insights into various aspects such behavior, trophic relations, feeding habits, dietary analysis, coprophagy, digestive tract structure and function, palynology, bacteria, parasitism, and organic geochemistry, including DNA and lipids.^13^^,^^14^^,^^15^^,^^16^^,^^17^^,^^18^^,^^19^^,^^20^^,^^21^^,^^22^^,^^23^^,^^24^^,^^25^^,^^26^^,^^27^^,^^28^^,^^29^^,^^30^ Coprolites have been recorded from throughout the Phanerozoic and from all continents since the 19th century. While the introduction of ichnotaxonomy for coprolites in 1998 was met with controversy by some workers,^7^^,^^31^ it has become an important classification method. Although the spiral coprolites have received considerable attention in previous studies,^28^^,^^32^^,^^33^^,^^34^^,^^35^ there has been a dearth of research on crocodilian coprolites.

The study of crocodilian coprolites can be traced back to 1832 (see also Table S1 for a comprehensive list). In a significant contribution, Robert^36^ reported several coprolites from the Eocene period in France and suggested their crocodilian origin. Over the course of the last two centuries, a total of 26 studies have documented crocodilian coprolites from 15 countries, spanning a wide range ages from the Early Cretaceous to the Holocene. The earliest potential crocodilian coprolites were discovered in Belgium*,*^37^^,^^38^ while the most recent findings pertain to *Crocodylus niloticus* in the Sahara Region, Africa*.*^39^ Among these coprolite localities, nine are from the Mesozoic era, comprising eight from the Cretaceous period and one from the Triassic (Crocodylomorph). The abundance of crocodilian coprolite localities is more pronounced during the Cenozoic era, with 16 reported instances. Notably, 11 of these localities date back to the Paleogene era, including the Na Duong coal mine and the Nanxiong site (Paleocene) in China, which has been extensively investigated by Young.^40^

**Figure 1:**
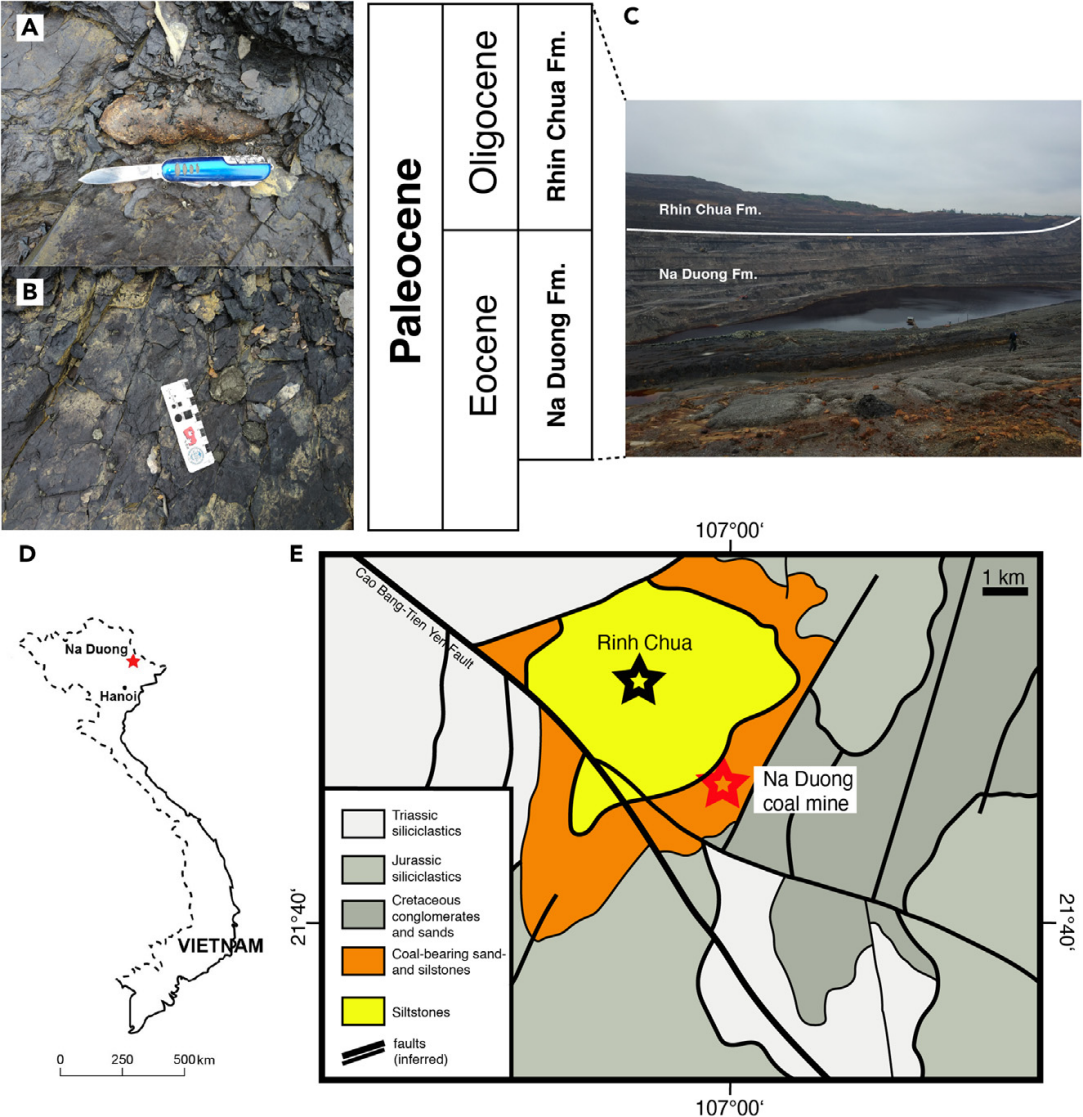
Locality where the coprolites were retrieved (A) *In situ* coprolites at Na Duong coal mine. (B) The coprolites were well-preserved in a largely and dense manner. Scales as indicated in figure. (C) Overview of the Na Duong coal mine. (D) Map of Na Duong Basin in Lang Son Province, Northern Vietnam. (E) Simplified geological map of the Na Duong basin (derived from^115^^,^^118^). Scales as indicated in the diagram.

In this study, we present a comprehensive analysis of 55 exceptionally well-preserved coprolites, attributed to crocodilians, from the Late Eocene deposits of Na Duong Formation in Lang Song Province, Northern Vietnam (Figure 1). The identification of a new ichnospecies contributes significant insights into the paleobiology of crocodilians in the Na Duong coal mine. Moreover, this research represents the first quantitative analysis of crocodilian coprolites from the Eocene epoch, thus shedding light on the morphological variations observed in the Na Duong coprolites and also the factors that contributed to their unusual preservation. By adopting a multidisciplinary approach, we emphasize the importance of trace fossils in deciphering paleobiological and paleoecological records. Furthermore, the study aims to reconstruct a snapshot of the Oligocene-Eocene environment of the Na Duong region, enabling us to infer potential factors contributing to the disappearance of its ecosystem.

## Results

### Systematic ichnotaxonomy

The formal ichnotaxonomy for vertebrate bromalites, or fossilized feces, has gained recognition relatively recently, with inconsistent utilization despite the long-standing acknowledgment of the necessity for naming trace fossils, as emphasized by Bertling et al. (^41^, p.265) for several decades. A notable example of the widespread acceptance and demonstrated value of vertebrate trace fossils lies in the realm of fossil footprints.^29^ The earliest instances of informal naming of coprolites can be traced back to the works of Buckland.^1^^,^^42^ Subsequent studies by Duffin,^43^ Laojumpon et al.,^44^ Milàn et al.^45^ and numerous others have exemplified the usage and utility of the ichnotaxonomy in classifying vertebrate bromalites. However, the reluctance to adopt a binomial scheme for bromalites (coprolites) can be attributed to the occurrence and misconception that taxa of modern feces are indistinguishable due to their variability, despite compelling evidences that extant animals can differentiated based on their excrement.^46^^,^^47^^,^^48^^,^^49^ Nevertheless, it is essential to consider the perspectives of certain researchers, such as Chin (in^50^) who choose to avoid such nomenclature for coprolites primarily due to two reasons: (1) the variable preservation of coprolites owing to taphonomy and diagenesis, and (2) that significant influence of dietary variations in producing distinct morphologies of feces for the same producer.

In this study, we adhere to the principles outlined by Hunt and Lucas,^7^^,^^50^ which strongly advocate for the use of formal nomenclature in the ichnotaxonomy of vertebrate coprolites, as it significantly enhances their utility in various fields such biostratigraphy, biogeography, and paleoecology, in comparison to relying solely on morphotypes.^7^ The International Commission on Zoological Nomenclature (ICZN)^51^ stipulates specific rules for trace fossil nomenclature. Since trace fossils represent the behavioral activities of organism rather than their actual remains, they are assigned ichnogenera and ichnospecies names, even though assigning coprolites to a specific producer at the family or higher taxonomic level^7^ is often challenging. Furthermore, ICZN guidelines specify that trace fossil should not be named using the same binomial as the organism that produced them.

For instance, Chame,^49^ a wildlife biologist, collected extant feces and developed a classification system that correlates feces with their respective producers. Similarly, Hunt and Lucas^50^ assigned binomial names to coprolites of *Mammuthus* and the extinct *Hyaena* that reflect their respective producers, namely *Mammuthocopros* and *Hyaenacoprus.*^50^^,^^52^ Based on these foundations, we adopt Hunt and Lucas’^50^ concept of coprolite nomenclature.

In this study, we introduce a novel nomenclature for crocodilian coprolites for the first time, given their distinct morphologies and unique structures. However, it is important to acknowledge that we fully aware of the ICZN rules and, accordingly, strongly advocate for the possibility of naming coprolites after the organism that produced them.

**Figure 2:**
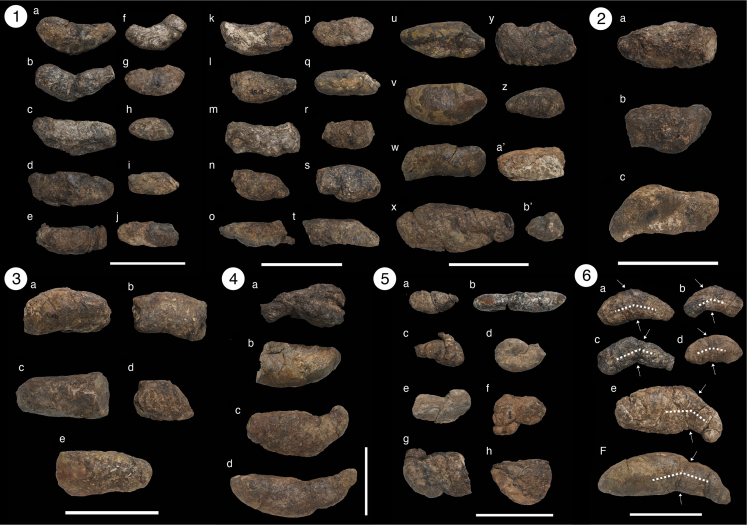
Morphotype plates of *Crococopros naduongensis* igen. et isp. nov **Plate 1**, Morphotype A; **Plate 2**, Morphotype B; **Plate 3**, Morphotype C; **Plate 4**, Morphotype D, whereby 4c is the holotype specimen; **Plate 5**, Morphotype U, and; **Plate 6** shows example of circumferential constriction marks. Scale bars equal 10 cm.
(urn:lsid:zoobank.org:act: 11743765-F8EA-419F-98DA-03B5DE7D9958)
  **Holotype.** IVPP V 27941/46 (Figure 2, Plate 4c)
  **Referred specimens.** IVPP V 27941/1 to IVPP V 27941/55 (Figure 2, Plate 1 to 4; see also Figure S2)
  **Type locality.** Na Duong coal mine, Northern Vietnam.
  **Type horizon.** Na Duong Formation.
  **Etymology.** The species name *naduongensis* is derived from the locality name, Na Duong, where the holotype specimen was found.
  **Distribution.** Same as the ichnogenus.
  **Description.** The holotype measures 201 mm in length with a maximum diameter of 58 mm and a second diameter of 42 mm. It exhibits a flattened end and a tapered end with a circular cross-section (Figure 2, Plate 4c). The coprolite displays a circumferential constriction mark that corresponds to a bend at an angle of approximately 120°–150° (Figure 2, Plate 6e), resembling extant crocodilian feces (^55^, Figure 1). The surface of *Crococopros naduongensis* igen. et isp. nov. is covered by a fragile, thin layer, and the interior appears massively homogenized. Some specimens consist of fused concavo-convex fecal units separated by thin layers. No visible surface borings, bite marks, or desiccation cracks are present. Wrinkled and rugose surface textures are common in all *Crococopros naduongensis* igen. et isp. nov. coprolites.

**Figure 3:**
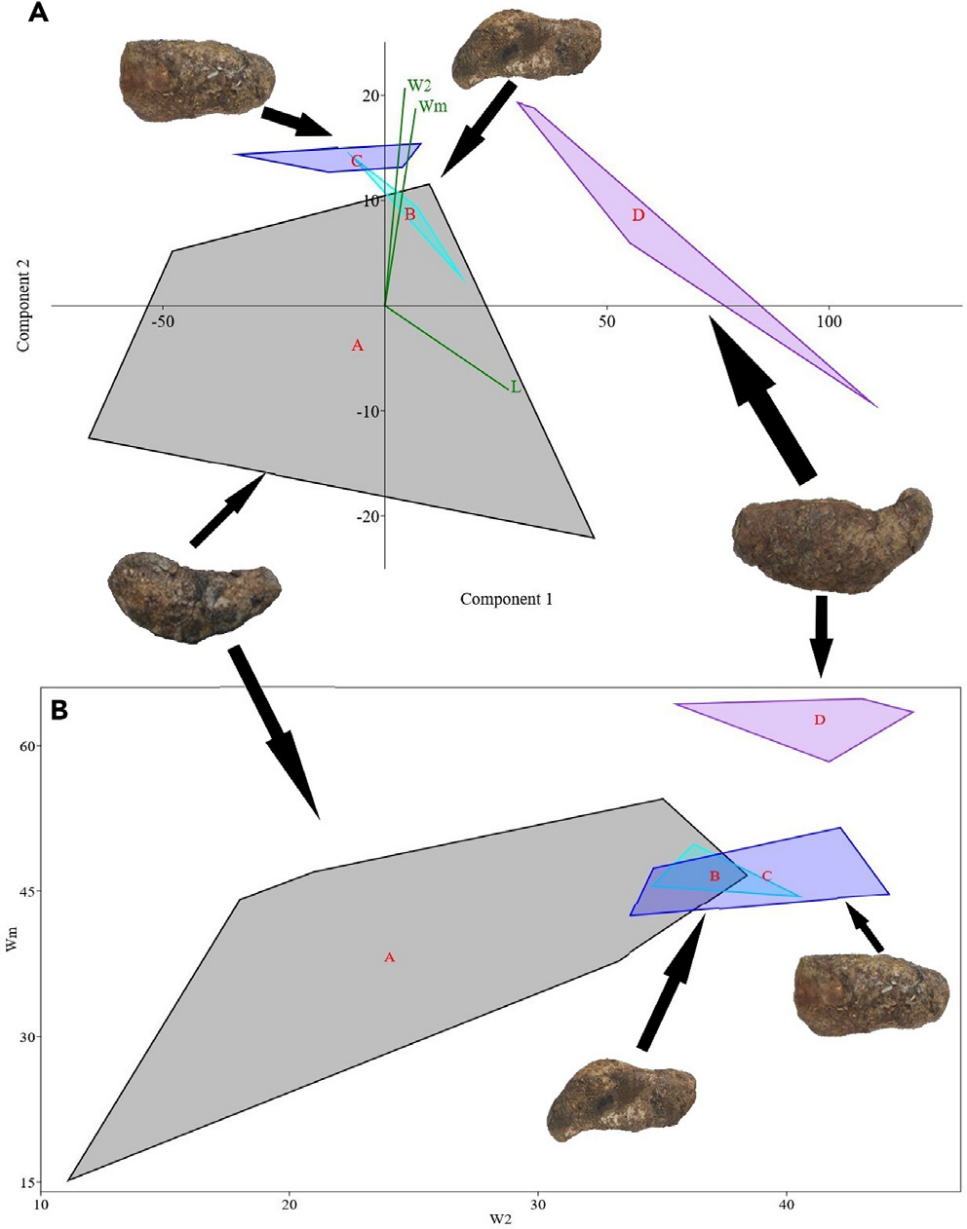
Quantitative analysis of *Crococopros naduongensis* igen. et isp. nov (A) Principal component analysis of variations of *Crococopros naduongensis* igen. et isp. nov. Diagram showing relationship between length (L), maximum width (Wm) and second width (W2) for *Crococopros naduongensis* igen. et isp. nov. morphotypes. (B) Maximum width (Wm) and second width (W2) distribution of morphotypes of *Crococopros naduongensis* igen. et isp. nov. The alphabet A, B, C, and D in the diagrams indicates subsequent morphology they belonged to.

**Figure 4:**
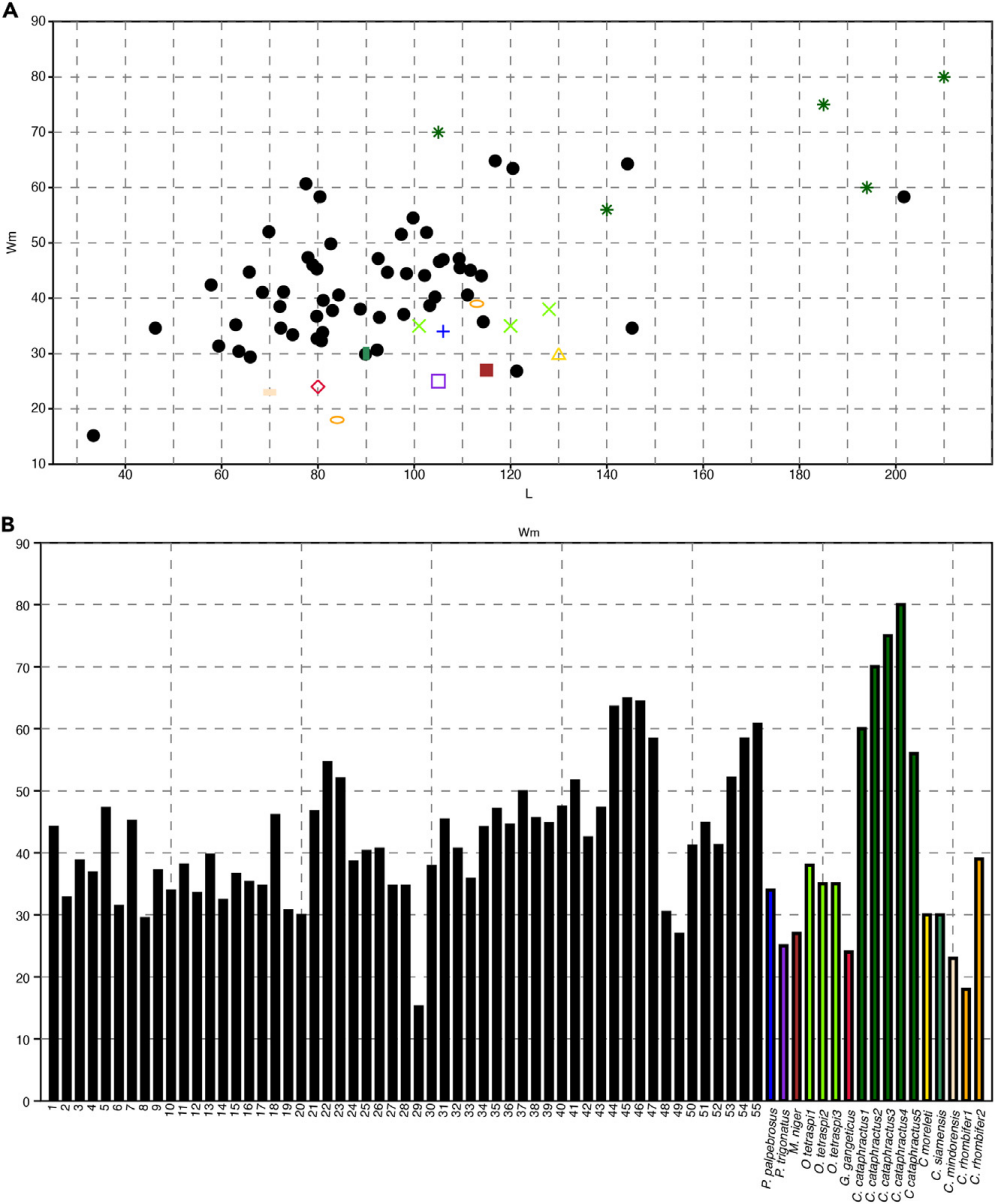
Comparisons between extant crocodilian feces and *Crococopros naduongensis* igen. et isp. nov (A) Maximum width distribution of extant crocodilian feces and *Crococopros naduongensis* igen. et isp. nov. No 1 to 55 refers to the number of coprolites from Na Duong coal mine. (B) Maximum width (Y axis) and length (X axis) distribution of extant crocodilian feces and *Crococopros naduongensis* igen. et isp. nov. Diagram symbols denoted as **Dot (Black)**: *Crococopros naduongensis* igen. et isp. nov.; **Star (Darkgreen)**: *Crocodylus cataphractus*; **Plus (Blue)**: *Paleosuchus palpebrosus*; **Square (Blueviolet)**: *Paleosuchus trigonatus*; **Filled square (Brown)**: *Melanosuchus niger*; **X (Chartreuse)**: *Osteolaemus tetraspi*; **Diamond (Crimson)**: *Gavialis gangeticus*; **Triangle (Gold)**: *Crocodylus moreleti*; **Bar (Seagreen)**: *Crocodylus siamensis*; **Dash (Bisque)**: *Crocodylus mindorensis*; **Oval (Orange)**: *Crocodylus rhombifer* (Extant crocodilian feces data from Milàn, 2012^55^).

*Crococopros***igen. nov.** Halaçlar et al., 2023 (This article).
  **Type species.***Crococopros naduongensis* isp. nov.
  **Included species.** Currently known only from the type ichnospecies.
  **Etymology.** The generic name *Crococopros* is derived from the Latin *crocodilus* for the presumed producer of the coprolites, and the Greek *kopros* for “dung” (the letter *k* has been altered to *c* as is customary in coprolite naming).
  **Distribution.** Upper Bartonian – Late Priabonian (34–39 MA), late Middle to Late Eocene of Na Duong coal mine, Northern Vietnam*.*^53^
  **Diagnosis.***Crococopros* coprolites are fusiform with cross sections oval in shape, featuring smoothly concave ends and circumferential zones of constriction. They lack longitudinal striations, distinguishing them from *Alococopros*. Additionally, *Crococopros* is larger than *Alococopros* and lacks the narrowly spaced “ribs” characteristic of *Costacoprus*. Compared to *Eucoprus*, *Crococopros* exhibits larger size, more rounded apices, and possesses circumferential zones of constriction, thus distinguishing them from all three mentioned ichnogenera.
  **Discussion.** Previous studies by Hunt et al.^54^ and Krause and Piña^12^ have noted fusiform, oval cross-sectioned crocodilian coprolites with smoothly concave ends. Similarities between some extant crocodilian feces and coprolites have been demonstrated by Milàn,^55^ supporting their attribution to crocodilian origin. While Alococopros was reported to have longitudinal striations by Hunt et al.,^54^ Milàn^55^ did not mention these features in extant crocodilian feces, suggesting the similarity to *Crococopros* in the absence of longitudinal striations. CT scans revealed fragmentary remains that could be associated with bone fragments, indicating a strong digestive system capable of bone destruction, which is a characteristic of a crocodilian origin.^12^^,^^54^^,^^55^ The presence of circumferential zones of constriction in *Crococopros* aligns with the descriptions by Milàn et al.^45^ of circumferential constriction marks on extant crocodilian feces and some coprolites from the Middle Eocene to the earliest Oligocene of Denmark. Similarly, Krause and Piña^12^ also observed a similar feature in crocodilian coprolite (^12^, Figures 5.3 and 5.4). Notably, *Crococopros* lacks the longitudinal striations present in *Alococopros*, and the size of most *Crococopros* specimens is larger than *Alococopros*. *Costacoprus* is characterized by narrow transverse “ribs” with close rounded spacing (^56^, Figures 2R and 2S), which is distinct by from the features seen in *Crococopros*. In comparison to *Eucoprus* (^57^, Figure 4; ^58^, Figures 3E–3V), *Crococopros* is larger in size and exhibits circumferential zones of constriction.

It is worth mentioning that Young’s^40^ coprolites display morphologically and biometric similarities to *Crococopros naduongensis* igen. et isp. nov. The coprolites from Young’s study, found in the Nanxiong Basin, Guangdong Province, South China, share similarities in morphology and biometry with the Na Duong coprolites., Both localities belong to the Paleogene and have recorded three different crocodilian species concurrently. Further analysis, including biogeochemical and biometric data from the Nanxiong coprolites, is necessary to support this hypothesis.

*Crococopros naduongensis***isp. nov.** Halaçlar et al., 2023.
(Figure 2, Plate 1 to 4; Figure S2)

The sediment color (light yellowish brown, HEU 2.5YR 6/3; dark gray, HEU 2.5YR 4/1 and dark, HEU 2.5YR 2.5/1) and the coprolite color (dark olive brown (HUE-5Y 3/3) are similar, appearing as light olive brown 341 (HUE-2.5Y 5/6)). The interior color of the coprolites is mostly olive brown (HUE 10YR 4/5) with some light gray patches (HUE 10years 7/1), which is lighter than the coprolite surface and the surrounding matrix. No signs of abrasion were observed on any of the coprolites, indicating good preservation. The uniform coloration suggests that all the coprolites were buried under similar sedimentary conditions. There is no evidence of diagenetic flattening, and most coprolites are well-preserved.

The coprolites exhibit variations in size (see also Table S2). The smallest complete specimen, IVPP V 27941/29, measures 33 mm in length and 29 mm in maximum width, while the largest complete specimen, IVPP V 27941/47, measures 200 mm in length and 57 mm in maximum width. The holotype, IVPP V 27941/46, measures 144 mm in length and 64 mm in maximum width. Incomplete specimens display various sizes and shapes, likely due to structural deformation resulting from the plasticity nature of expelled feces. Quantitative analyses were conducted using only complete or nearly complete specimens, while fragmentary crocodilian coprolites were used for destructive analyses.

Chin^59^ suggested that recurring size classes in a large sample of coprolites from a given locality may indicate different taxa or age classes. *C. naduongensis* igen. et isp. nov. can be classified into five morphotypes based on their morphology (Figure 2, Plate 1–5; see also Table S3).

#### Morphotype A (MorA)

This is the most abundant type of *C. naduongensis* igen. et isp. nov. with a total of 35 specimens. The average length of MorA is 88 mm, and the average maximum diameter is 38 mm. It is a medium-sized coprolite, slightly flattened in shape, with one end more tapered than the other (Figure 2, Plate 1).

#### Morphotype B (MorB)

This morphotype is characterized by its large size and flattened shape, with both ends tapered. The number of specimens for MorB is 3. It exhibits greater differences between the maximum diameter and the second diameter compared to other variations (Figure 2, Plate 2).

#### Morphotype C (MorC)

This is the second most abundant type of *C. naduongensis* igen. et isp. nov. with a total of 5 specimens. MorC has an average length of 84 mm, an average maximum diameter of 46 mm, and an average second diameter of 39 mm. It is oval in shape, with a circular cross-section, and both ends are tapered (Figure 2, Plate 3).

#### Morphotype D (MorD)

These coprolites are large and massive, with one end significantly more tapered than the other. The number of specimens for MorD is 4. They have a circular cross-section and exhibit a well-developed circumferential constriction mark. The average length of MorD is 148.5 mm, the average maximum diameter is 63.5 mm, and the average second diameter is 46 mm (Figure 2, Plate 4).

#### Morphotype U (Uncategorized group)

This group consists of eight specimens and includes one specimen initially thought to be a regurgitalite. However, CT scan observations did not reveal any residues, suggesting taphonomic distortion. Therefore, we consider it as a taphonomically distorted coprolite (Figure 2, Plate 5).

#### Discussion

*C. naduongensis* igen. et isp. nov. exhibits morphology and size comparable to feces of extant crocodilians (^12^, Figure 6—6; ^55^, Figure 1), as highlighted by Milàn’s^55^ study, which involved the collection of 17 fecal samples from 10 different crocodilian species. These findings provide insights into the variations observed in *C. naduongensis* igen. et isp. nov.

In their previous work, Böhme et al.^53^ described the crocodilians from Na Duong formation and identified three morphotypes. The brevirostrine morphotype, constituting 2/3 of the Na Duong crocodilians, measures around 2 m in length. The longirostrine crocodilian morphotype, representing 1/3 of the Na Duong crocodilians, exceeds 6 m in length. Additionally, a second specimen from the Na Duong coal mine belongs to the longirostrine gavialoid type. Milàn^55^ argued that the feces of *Gavialis gangeticus*, a longirostrine gavialoid, would be unlikely to be preserved due to their tendency to disperse after defecation in water bodies. Therefore, we infer that *C. naduongensis* igen. et isp. nov. was most likely produced by the brevirostrine or longirostrine crocodilian types (see also Table S4).

The brevirostrine type crocodilian, which is the smallest and the most abundant crocodilian in the Na Duong herpetofauna, is likely associated with MorA, the smallest and the most abundant morphotype of Na Duong coprolites. Despite its larger size, MorB shares a flattened cross-section similar to MorA. Consequently, we deduce that MorB was produced by the large-sized brevirostrine type crocodilian, as supported by the presence of only four specimens.

Biometric analysis using component analysis and Wmax/Wsec plot diagrams (Figures 3A and 3B; see also Table S5) clearly differentiates the morphotypes of *C. naduongensis* igen. et isp. nov. MorD and MorA occupy distinct positions on the biometric scale, while MorB and MorC appear closer to MorA due to the wide range of MorA’s measurements. The primary distinctions between MorB and MorC lie in their Wmax and Wsec ratios, with MorC exhibiting a cylindrical cross-section and MorB an oval cross-section. Previous studies have suggested that the diameter of coprolites is more informative than their length.^59^ Our study provides a comparative analysis through component analysis and plot diagrams, both of which demonstrate the distinguishability of *C. naduongensis* igen. et isp. nov. (Figures 3A and 3B). MorD, being the largest type of *C. naduongensis* igen. et isp. nov. coprolite, is likely produced by the longirostrine crocodilian type measuring over 6 m in length. Figure 4 illustrates that a 450 cm-long *Crocodylus cataphractus* can generate a minimum focal length of 5 cm, while a 250 cm-long extant crocodilian can only produce feces with a maximum diameter of 4 cm. Considering MorC exceeds 4 cm in length, it is plausible that the longirostrine crocodilian could be the producer. Additionally, both MorC and MorD exhibit circular cross-sections, sugsting that MorC could be associated with a juvenile longirostrine crocodilian.

Milàn’s^55^ work presented evidence that a 450 cm-long *C. cataphractus* can produce feces wider than 4 cm. Platt et al.^60^ conducted a quantitative analysis on 41 fecal samples from *Crocodylus siamensis* and *Alligator mississippiensis*, demonstrating that *C. siamensis* measures less than 200 cm in total length can only produce feces with a maximum diameter of 2.5 cm (^60^, Figures 2, 3, and 4). Similarly, *A. mississippiensis* measuring less than 200 cm in total length can barely produce any feces exceeding 4 cm in diameter (^60^, Figures 3 and 4). With this information and referring to Figure 4, we deduce that some specimens of *C. naduongensis* igen. et isp. nov. specimens with a diameter larger than 4 cm were likely produced by the longirostrine crocodilian type.

#### Copro-biostratigraphy and copro-biochronology of archosaurs

In recent years, several studies on archosaur coprolite have emphasized the significant of trace fossil records. At the outset of this study, we introduce the term “copro-phylogeny”. Copro-phylogeny refers to the phylogenetic examination of coprolites using cladograms to identify ichnogenera and ichnospecies. While phylogeny traditionally involves understanding the origins and relationships among species (branching), this direct approach is challenging in the field of trace fossils. These difficulties hinder the development of suitable character matrices for distinguishing vertebrate coprolites. Currently, the most viable method for comparison involves copro-biostratigraphy and copro-biochronology, which utilize morphological features and correspondences to the probable producers of known vertebrate coprolites. However, we believe that as more coprolite ichnotaxonomy work is conducted in the future, unique findings regarding coprolites will emerge.

**Figure 5:**
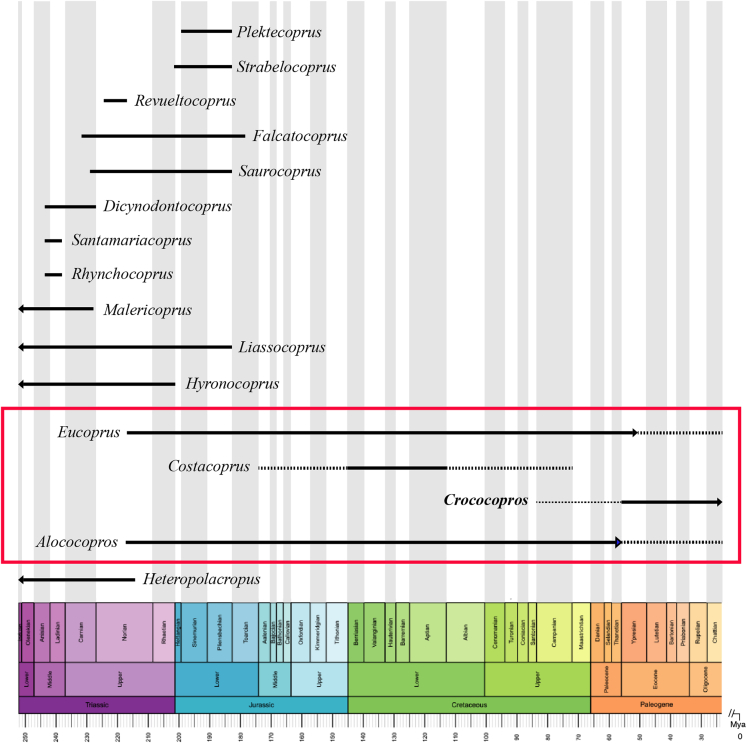
Vertebrate coprolite biostratigraphy and biochronology of *Crococopros* in related to other coprolite ichnogenera in the Phanerozoic record Box in red shows the biochronology of *Alococopros*, *Eucoprus*, *Costacoprus* and *Crococopros* (added and revised from^7^).

At present, in a preliminary manner, we propose a revision for the ichnogenus *Eucoprus* (Figure 5). This revision is prompted by the observation made by Hunt and Lucas,^56^ suggesting that *Eucoprus* may have been produced by basal archosaurs during the Mesozoic. Subsequently, similar coprolites were discovered in the Eocene of Kazakhstan, and these specimens were classified under the same ichnogenus. In this study, we argue that the Eocene specimens from Kazakhstan likely belong to *Crococopros* due to their resemblance to coprolites found in small sized crocodilians from the Eocene of Kazakhstan. On the other hand, *Costacoprus* and *Alococopros* exhibit distinct morphologies, suggesting that they were produced by other reptilian species other than crocodilians. However, a detailed comparison of these ichnogenera will not be discussed in this particular study.

#### Neocoprological study

The fossil coprolites exhibit clear morphological similarities, in terms of both shape and size, with the feces of *Crocodylus* *siamensis* (see also Figure S3) and *C. naduongensis* igen. et isp. nov. The largest feces collected from the farm, belonging to the extant crocodilians, measured 93 mm in length and 36 mm in width. Morphologically, these modern feces have rounded ends, with one of them displaying a circumferential constriction mark. Additionally, they are frequently covered by a thin layer (ranging from 1 to 5 mm) that is darker in color compared to the groundmass of coprolites. Longitudinal striations are absent on these feces. Moreover, the fresh surface of the feces tends to have numerous sand particles adhering to it, which can cause the outer layer to easily dissociate and detach. This present challenges for study purposes, although collecting the feces immediately after defecation poses certain risks. In some cases, broken feces can provide insights into their internal structure, occasionally revealing inclusions such as chicken feathers, but no bone fragments have been observed thus far. Observations conducted at the Beijing Zoo have shown that crocodiles exhibit defecation behaviors both in water bodies and on terrestrial surfaces, depending on their disposition.

### Analyses

#### Histological thin sections and petrography

**Figure 6:**
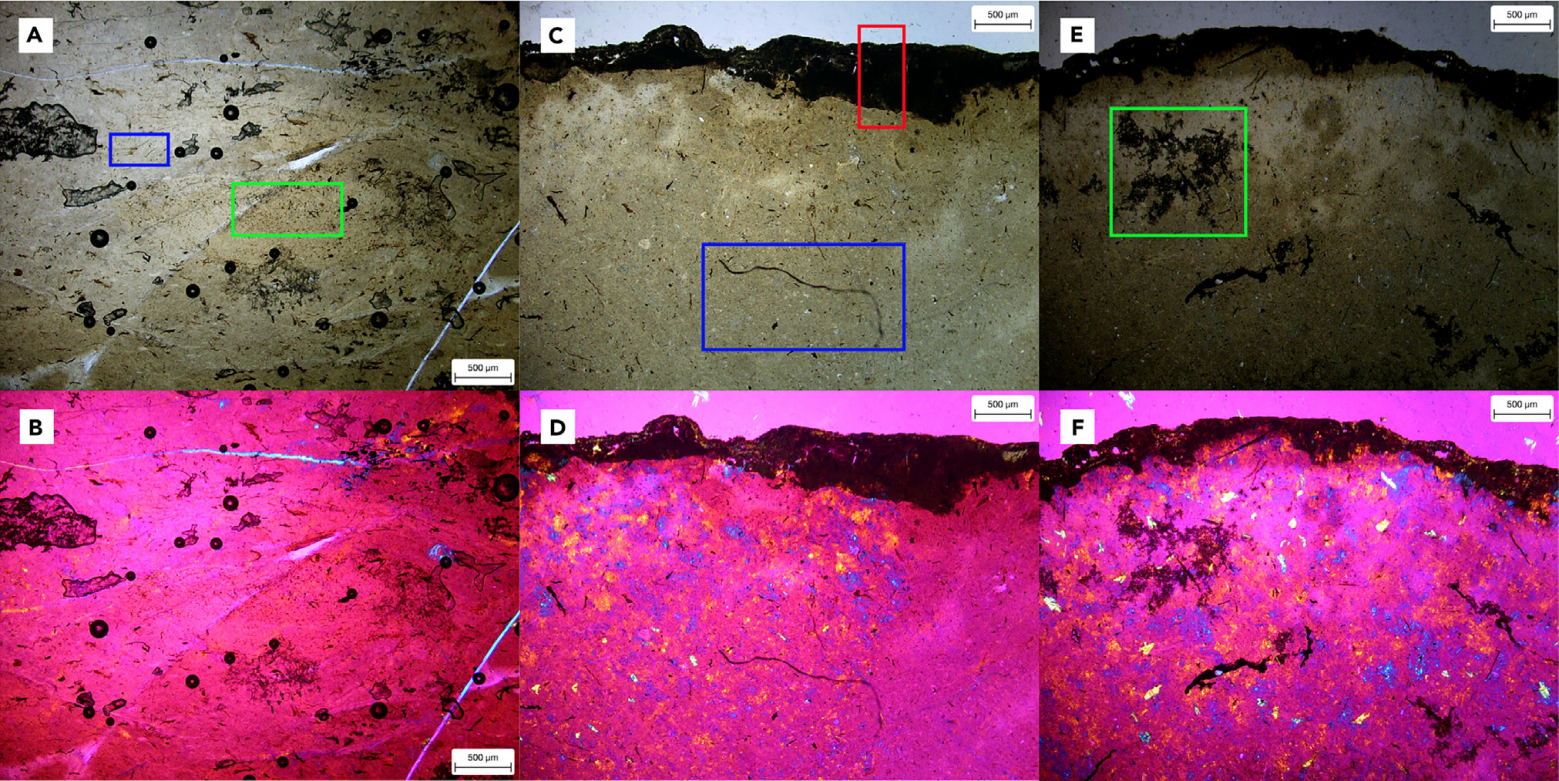
Histological characteristics of *Crococopros naduongensis* igen. et isp. nov. under normal light and cross-polarized light with lambda compensator (A and B) were from specimen IVPP 27941/3; (C and D) were from specimen IVPP 27941/45; and (E and F) were from specimen IVPP 27941/49. Green boxes indicate possible soft-tissues or organic matters preservation; red box indicates the superimposed layer of the coprolite; blue boxes indicate filamentous elements. Scales as indicated in diagrams.

The examination of histological thin sections and petrography provide evidence suggesting the preservation of soft-tissues and other organic matter in the coprolite samples, although further study is needed to investigate this in more detail. Notably, a superficial thin mucosal layer is clearly visible in the three coprolite samples (Figure 6). However, when thin sections of extant feces were analyzed, the results were not suitable for direct comparison. It is advisable to consider comparing the thin sections of coprolites with feces collected from wild crocodiles, as they may provide a more appropriate basis for comparison than feces from farms.

#### Inclusions

**Figure 7:**
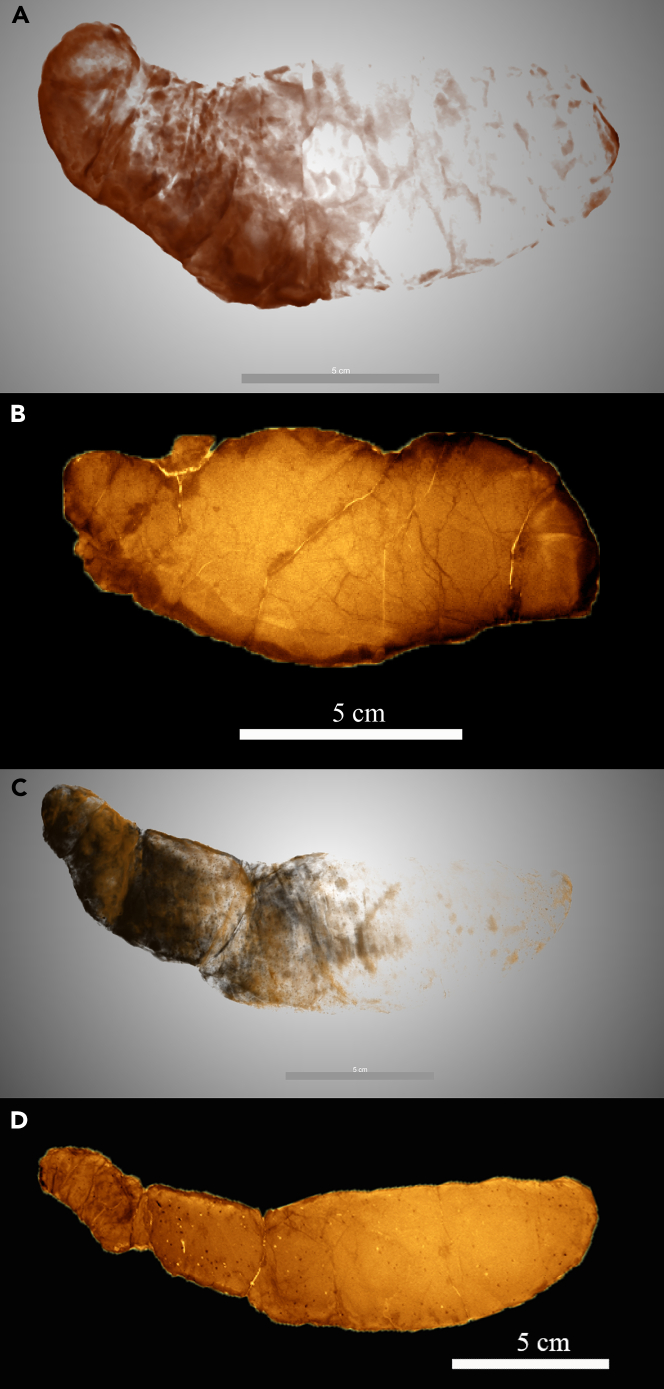
Diagrams showing CT scan images of *Crococopros naduongensis* igen. et isp. nov. coprolites (A) CT scan rendering of holotype specimen IVPP V 27941/46. (B) CT slice image of holotype specimen IVPP V 27941/46. (C) CT scan rendering of referred specimen IVPP V 27941/47. (D) CT slice image of referred specimen IVPP V 27941/47. Dragonfly Pro Workstation was used for producing the above images. Colors are altered for better effects and visualization. Scales as indicated in respective images.

Upon conducting CT scans observations, it became evident that specimen IVPP V 27941/46 and IVPP V 27941/47 did not contain any detectable residues (Figure 7). However, specimens IVPP V 27941/3, IVPP V 27941/36, IVPP V 27941/40, IVPP V 27941/49, IVPP V 27941/51, and IVPP V 27941/53 exhibited a small number of fragmented residues. The infrastructures in these specimens appear colorized as light gray (HUE 10years 7/1) and olive brown (HUE 10YR 4/5). Additionally, with the exception of IVPP V 27941/46, tiny holes are visible in all other scanned specimens. No internal borings were observed during the examination. Notably, circumferential constriction marks are clearly visible in the slicing images.

### SEM-EDS analyses

All of the coprolite samples exhibited significant peaks of carbon (C), calcium (Ca) and phosphorus (P). In addition, IVPP V 27941/47 and IVPP V 27941/49 displayed peaks of oxygen (O) and iron (Fe), whereas IVPP V 27941/3 did not exhibit these peaks. The sediment sample, on the other hand, yielded peaks for potassium (K), oxygen (O), iron (Fe), sodium (Na), aluminum (Al), silicon (Si), and sulfur (S). A comprehensive summary of these analyses can be found in the supplementary data (see also Table S6).

### Palynology

**Figure 8:**
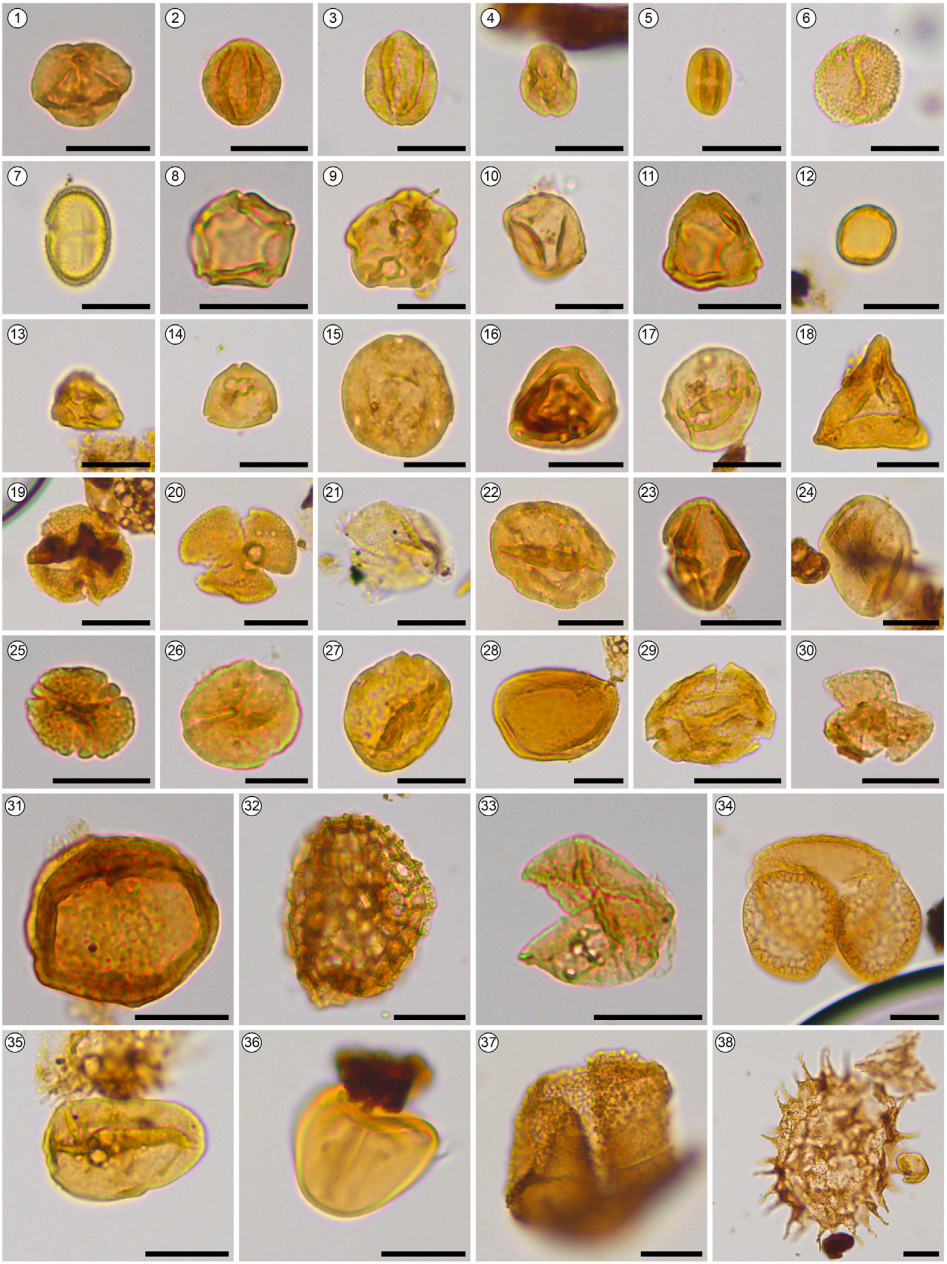
Results yielded from palynology studies Common palynomorphs of coprolites and their surrounding rocks in Na Duong coal mine. **1–4,***Quercoidites*; **5,***Cupuliferoipollenites*; **6,***Sparganiaceaepollenits*; **7,***Rutaceoipollenites*; **8,***Alnipollenites*; **9,***Pterocaryapollenites*; **10,***Carpinipites*; **11,***Betulaepollenites*; **12,***Moraceoipollenites*; **13,***Platycaryapollenites*; **14,***Engelhardtioidites*; **15,***Caryapollenites granulatus*; **16,***Caryapollenites polarannulus*; **17,***Celtispollenites*; **18,***Cupanieidites*; **19, 26, 30,***Tricolpites*; **20,***Shorea*-Type; **21,***Retitricolpites/Tricolpites*; **22,***Liquidambarpollenites*; **23,***Tricolporopollenites*; **24,***Cornaceoipollenites*; **25,***Operculumpollis*; **27,***Ulmipollenites*; **28,***Monocolpopollenites* (Arecaceae); **29,***Nothopollenites*/*Fupingopollenites*; **31,***Nymphaeacidites*; **32,***Persicarioipollis*; **33,***Taxodiaceaepollenites*; **34,***Pinuspollenites*; **35,***Polypodiaceaesporites*; **36,***Deltoidospora*; **37,***Osmundacidites*; **38,***Pediastrum*. (Scale bar equals 20 μm).

In this study, a total of 4 coprolites with surrounding matrices were subjected to palynological analysis, and all samples yielded abundant pollen grains. The palynological analysis identified a total of 76 palynomorph types, comprising 5 gymnosperms, 66 angiosperms, 3 ferns, and 2 algae (Figure 8; see also Table S7). Interestingly, the palynomorphs found in the coprolites were identical to those present in the surrounding rocks. However, the palynological concentration in the surrounding rocks (5100 grains/g) was higher than that in the coprolites (300 grains/g). The dominant palynological assemblage was composed of angiosperms, accounting for approximately 92.8–98.9% (average 96.6%). Gymnosperms constituted a smaller proportion, ranging from 0.8 to 5.4% (average 2.2%), while ferns represented around 0.2 to 3.9% (average 1.2%). Within the angiosperms, the most prevalent species was *Quercoidites*, constituting 39.8 to 62.5% (average 54.4%), followed by *Operculumpollis* (6.3%), *Momipites* (4.8%), *Liquidambarpollenites* (4.1%), *Caryapollenites* (2.0%), and *Ulmoideipites* (2.0%). Several less abundant but frequent species were also observed, including *Betulaceoipollenites*, *Carpinipites*, *Rutaceoipollenites*, *Euphorbiacites*, *Shorea*-type, Arecaceae, and *Fupingopollenites*. Among the gymnosperms, *Taxodiaceaepollenites* dominated, accounting for approximately 2%, and occasionally, *Abietineaepollenites*, *Pinuspollenites*, *Podocarpidites*, and *Ephedripites* were also present. The ferns mainly consisted of *Osmundacidites*, *Deltoidospora*, and *Polypodiaceaesporites*. Additionally, a considerable quantity of freshwater plankton algae colonies was discovered, mainly represented by *Pediastrum* sp., with a concentration ranging from 170 to 6480 grains/g. It was observed that the concentration of algae colonies in the sediment was higher than that in the coprolites. For a comprehensive list of the palynomorphs identified during the palynology analysis on *C. naduongensis* igen. et isp. nov. specimens and surrounding rocks from Na Duong formation are available in the supplementary data (see also Table S7).

## Discussion and interpretation

Determining the possible producer of coprolites poses significant challenges, especially considering their morphological variation and preservational conditions. The methods employed to infer their origin are complex and encompass various approaches, including stratigraphy, geographical relationships, chemical analysis, and neocoprology studies.^28^^,^^29^^,^^57^^,^^61^^,^^62^^,^^63^ Coprolites often contain valuable dietary residues, making them akin to *Lagerstätte*, which are frequently underrepresented in the fossil record. It is widely acknowledged that certain coprolites serve as exceptional preservation sites, constituting Konservat-Lagerstätten.^7^^,^^64^^,^^65^^,^^66^^,^^67^

In this study, the availability of abundant coprolite materials allowed for a comprehensive analysis, necessitating a destructive approach to generate more extensive data. Several lines of evidence support the fecal origins of the Na Duong formation material: (1) overall morphology, (2) morphology and size, (3) presence of fecal matter inclusions, (4) high calcium and phosphate content, and (5) comparisons with neocoprology studies. Notably, bioerosional scars, borings, cavities, and radial/concentric cracks were not observed in most coprolites. The subsequent discussion will focus on the biogeochemical results, while refraining from repeating aspects previously addressed in the ichnotaxonomy section. It is important to emphasize that the interpretation of the coprolites’ crocodilian origin presented in the ichnotaxonomy section was strongly influenced by the results obtained here.

Due to the existence of four distinct morphologies among the coprolite specimens, inferring the possible producer based solely on size and shape is not feasible. However, there is a clear hypothesis regarding the producer, considering specimens of MorD, including the holotype specimen of *C. naduongensis* igen. et isp. nov. (IVPP V 29741/46), are all relatively large. Thus, the producer must be a massive animal since large animals are capable to produce small-sized excrement, whereas small animals cannot produce large-sized excrement.^18^^,^^68^

SEM-EDS analysis revealed high levels of calcium, phosphorus, and other organic materials in the coprolites. This suggest a carnivorous producer,^69^ although it is important to consider that the initial compositions often undergo alteration during the fossilization process.^23^ Herbivorous excrement generally lacks phosphates and relies more on other mineral enrichment for fossilization,^70^ making the preservation of herbivorous coprolite relatively uncommon.^71^ Considering the morphological shape and evidence from Krause and Piña,^12^ which showed similar high peaks of carbon (C), calcium (Ca), oxygen (O), and phosphorus (P) in EDS analysis of modern caiman feces, it can be inferred that these coprolites were inevitably produced by carnivorous organisms.

CT scan results indicated that bone residues in the scanned coprolites were fragmentary or absent. No internal borings were detected, but, small round tiny holes were present. Inclusions in coprolites not only provide insight into the producer’s diet but also help infer their digestive strategies, which are closely related to food intake and digestion processes (see^72^ for review). All the coprolites from Na Duong likely belong to the second digestive strategy, as the presence of fragmentary or no bone residues suggests a strong gastric motility in the producer’s stomach. This type of digestive strategy typically involves limited mastication, enhanced digestive assimilation, prolonged gut time for better nutrient absorption, and is commonly associated with crocodilian origins.^63^^,^^73^^,^^74^^,^^75^^,^^76^ The absence of inclusions due to an advanced digestive system supports this inference and is further supported by the paleoecology of the Na Duong formation, indicating that crocodiles likely preyed on large animals.^25^^,^^55^^,^^77^^,^^78^^,^^79^^,^^80^ Notably, numerous tiny holes or microvoids were visible in all the CT scanned materials, except for the holotype specimen IVPP V 27941/46, possibly caused by gasses trapped within the fecal matters during the producer’s digestion processes.^81^^,^^82^^,^^83^ If the fecal mass was excreted in water bodies,^84^ these microvoids could quickly fill with water. It is worth noting that none of the Na Duong coprolites exhibits breakage or radial and concentric cracks, indicating rapid burial before any coprophagy occurred. The absence of boring traces suggests that the coprolites were promptly buried, preventing coprophagy from taking place.

The mineral composition of coprolites, as determined by EDS analysis, shows a correlation with their color, which may be influenced by depositional factors.^12^ The light color of most of the Na Duong coprolite interiors is associated with the presence of calcium in carnivorous diets.^49^^,^^85^^,^^86^^,^^87^ Conversely, dark colors may be attributed to the presence of iron or complete phosphatisation.^12^^,^^43^ The presence of iron (Fe) is indicated in the elemental graphs (Table S6). However, it is important to note that diagenesis can significantly impact colorations.^12^^,^^27^ The dark colorization on the outer layer also suggests the presence of fresh mucosal layers that were superimposed before rapid burial.

Histological thin sections or petrography can provide valuable information about coprolite content, although thin section sampling can be unpredictable. Unlike body fossils, the identification of dietary components using this technique can be challenging due to selective feeding and random inclusions. Analysis becomes particularly difficult when coprolites have few inclusions, as is the case in our study. Thin sections analysis can offer insights into diagenetic mineralization. In our study, there may have been traces of soft tissue and organic matter, but their identification was hindered by the lack of solid comparable examples and available data. Filamentous structures are visible on thin section slides (Figure 6C). One notable observation is that all three thin sections exhibited similar structures, indicating that the coprolites originated from the same type of producer. There is a need to develop a proper protocol and comprehensive census of biomarkers to enable accurate identification processes.

While fossil and extant crocodilians are known to possess gastroliths, their occurrence in the fossil record is rare.^7^^,^^40^^,^^88^^,^^89^^,^^90^^,^^91^ No traces of gastroliths were found on the surfaces of the coprolite materials, including the scanned inclusions of selected specimens. It is important to acknowledge that the absence of gastroliths does not entirely rule out their presence in the coprolites, as gastroliths typically occur on the surfaces of coprolites^88^ and could have easily detached during taphonomic processes. Some hypotheses suggest that stones in crocodilians may serve buoyancy purposes, but such ideas are not convincing when considering the numbers of gastroliths in relation to the crocodile’s body size.^7^

The identification of pollen in coprolites is crucial for reconstructing paleoenvironment and gaining insight into ecosystem evolution.^92^^,^^93^^,^^94^^,^^95^^,^^96^ The presence of palynomorph assemblages in crocodilian coprolites and the stomach contents of extant crocodilians is commonly observed, as these palynomorphs are acquired during feeding or by ingesting the gut contents of prey, whether in a semi-aquatic or terrestrial environment.^97^^,^^98^^,^^99^^,^^100^ The palynological assemblage from the Na Duong coprolites indicates that the dominant species was the evergreen *Quercu*s, accompanied by subtropical tree species such as *Carya* and *Liquidambar*, as well as deciduous components like *Betula* and *Carpinus*. Additionally, pollen of Dipterocarpaceae (genus *Shorea*, Roxburg ex C.F. Gaertner), which are prominent in the tropical rainforests of modern South Asia and Southeast Asia, was also found in the samples. Therefore, the palynological assemblage suggests a vertical variation in vegetation near the Na Duong Basin in the Late Eocene. The upland area was characterized by a subtropical evergreen broad-leaved forest dominated by evergreen oaks, while the shore shore facies of lakes and swamps featured common tree species found in tropical rainforests, such as *Shorea* and palm. Numerous freshwater algae and aquatic plants, including algae, water lilies, and duckweed, were present in lakes and swamps. The palynological assemblages indicate a warm and humid tropical/subtropical climate during the Late Eocene in the Na Duong Basin.

It is widely recognized that coprolites can be transported from their original place of deposition through various modes, as evidenced by signs of abrasion.^101^^,^^102^ However, the Na Duong coprolites show no signs of abrasion, and most of them were preserved three-dimensionally with minimal damage. This supports our hypothesis that these coprolites were excreted in shallow water bodies, such as lakes or the river banks, where there was little turbulence or current.

Most Phanerozoic coprolites are found in low-energy shallow marine or lake environments,^18^ where fecal preservation is enhanced by rapid burial in humid conditions and the acidity level of the water bodies.^33^^,^^57^^,^^103^^,^^104^^,^^105^ In addition to these trophic factors, several crucial factors influence the fossilization of coprolites. The content and composition of the fecal matter itself are important criteria, with coprolites from carnivores diets constituting a larger proportion of the fossil record compared to herbivores.^59^ The taphonomic history of coprolites involves three main stages: before final burial, after final burial, and after exposure.^105^ In the case of the Na Duong coprolites, the third stage did not occur, as indicated by the well-preserved delicate mucosal layers on the outer surface. The exceptional preservation of the Na Duong coprolites suggests rapid burial in fine-grained sediments. Such favorable conditions are rare, making the taphonomy of Na Duong’s coprolites a remarkable event.

### Paleogeographical implication

The Nanxiong locality in Southern China, known for its Paleogene deposits, has yielded crocodilian coprolites believed to be from *Asiatosuchus nanlingensis* and *Eoalligator chuyii,*^40^ whose morphology is compatible with *C. naduongensis*. This suggests a strong possibility that all these specimens produced coprolites that can be assigned to the same ichnogenus ‘*Crococopros*’. Furthermore, Eocene crocodilian fossils, such as *Tomistoma petrolica*^106^ and an indeterminate Alligatorid*,*^107^ have been discovered in the Maoming Basin, indicating a diverse crocodilian fauna in the region. The presence of the genus *Shorea* in the Huangniuling flora (Maoming Basin) also suggests the existence of tropical forests in the area.^108^^,^^109^ Interestingly, our studies at Na Duong have also identified the presence of *Shorea*, and both localities share similar latitudes (Figure 1C). These paleographical features of flora and fauna in the two localities imply a similar ecosystem evolution and tropical weather events. For example, the Youganwo Formation (Maoming Basin) was initially a swampy lacustrine–fluvial plain that later transformed into a freshwater lake. Similar natural succession may have affected Na Duong and eventually led to the disappearance of its rich flora and fauna.

Other Eocene localities in southwest Asia, such as Pondaung (Burma) and Krabi (Thailand), not only exhibit similar paleoenvironments and paleoecology to the Na Duong Basin but have also yielded crocodilian and mammalian fossils.^53^^,^^110^^,^^111^^,^^112^ Tsubamoto et al.^110^ reported the discovery of reptilian bones, crocodilian teeth, coprolites, and gastropod molds in the Thandaung Kyit-chaung locality of the Pondaung Formation. Although their coprolite findings were not published, we strongly believe that these coprolites could be of reptilian origins, thus opening-up further paleogeographical connections.

Crocodilian are often used as paleoclimate proxy^113^ due to their sensitivity to climate variations. Markwick (^113^, Figure 3) demonstrated a peak in crocodilian generic diversity during the middle to late Eocene, followed by a sharp decline in the late Eocene. The Na Duong basin has yielded numerous crocodilian fossils and coprolites, aligning well with the middle to late Eocene age determined by Böhme et al.^53^ Please refer to supplementary data (see also Table S8) for additional comparisons of paleogeography among Eocene localities with crocodilian coprolites.

### Paleoecological interpretation and paleoenvironment of the Na Duong Basin

The Na Duong coal mine strata predominantly represent a wet forest swamp environment, encompassing swamp, lake-margin, and lacustrine facies associations.^114^ Palynomorph assemblages and sedimentological data from Wysocka et al.^115^ indicate that Na Duong was a freshwater basin characterized by stagnant or slow-flowing water under humid climatic conditions, with no evidence of aridity.^53^ This aligns with the absence of subaerial exposure on the coprolites. Wysocka et al.^115^ highlighted the similarity between the Na Duong and Rinh Chua Formations, notwithstanding the fact that *Graminidites* (herbaceous plants) and *Chenopodipollis* were found only in the Rinh Chua Formation. They further concluded that sedimentation in the Na Duong Basin occurred in a warm temperate-to-subtropical climate.

The discovery of *Bakalovia orientalis* fossils in Na Duong represent a semi-aquatic lifestyle^53^^,^^116^ common among buffalos and other semi-aquatic artiodactyls. Six of the eight *Bakalovia orientalis* specimens found in Na Duong were juveniles, indicating high juvenile mortality, potentially caused by crocodile predation.^53^ In contrast, *Epiaceratherium naduongense* exhibits slender and long limbs, characteristics of terrestrial animals, and possessed a dental formula and browsing habits resembling tapirs.^117^ These features indicate that they were forest-dwelling species.^53^ The presence of diverse crocodilians (three types) suggests the availability of abundant food resources and the presence of dense vegetation that would serve as suitable habitat for crocodilian ambush predation and breeding grounds.

Böhme et al.^118^ observed poorly preserved anatomical structures on the wood, indicating separation from the stump and abrasion prior to fossilization. This was interpreted as evidence of transport and allochthonous deposition. Coprolites typically require rapid burial without transportation, suggesting that the tree trunks were abraded and transported before being buried alongside with the coprolites. This indicates that the deposition in Na Duong was stable and rapid, punctuated by periodic floods that brought in large tree trunks from upstream.

**Figure 9:**
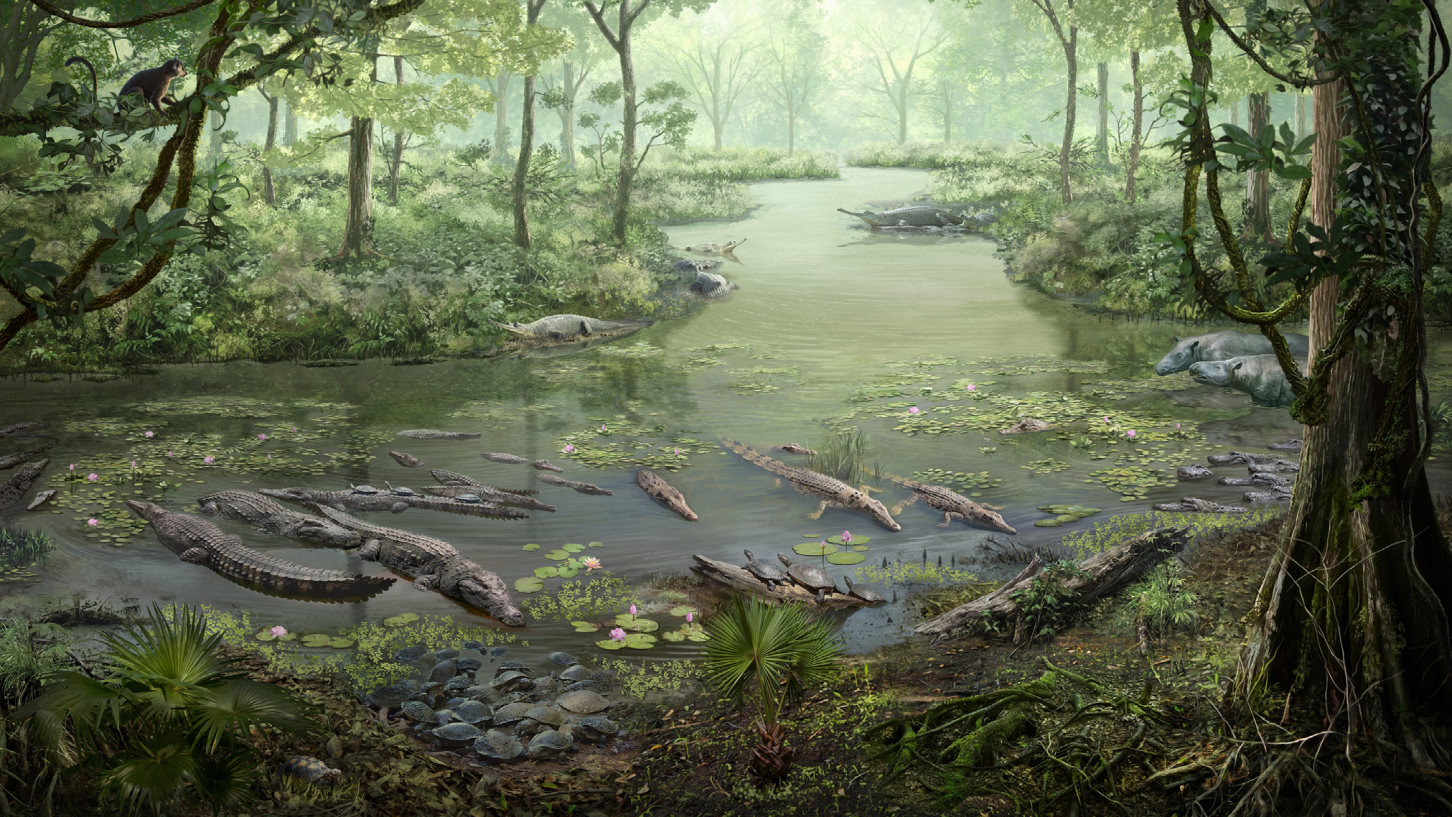
Paleoenvironment reconstruction drawing reflects a real ‘snapshot’ of an intermittently swamped lacustrine–fluvial plain ecosystem with an abundance of crocodilians and testudines The Eocene of Na Duong Basin is considered as a fossil Lagerstätte of Southeast Asia (Flora and fauna constituent in the drawing were an actual reconstruction based on palynology analyses, and of body fossil findings from various workers; illustration by Chung-Tat Cheung).

Figure 9 presents an idealized illustration depicting the reconstruction of the environment and paleoecology of the Na Duong coal mine.

### Limitations of the study

The majority of trace fossils research is subject to some limitation related to the objects of study. One limitation of this study is the assessment of morphological variability of the coprolites, which is largely influenced by taphonomic factors. These factors can affect the preservation and morphology of the coprolites, potentially leading to limitations in accurately characterizing their variability. Another limitation is the limited examination of the possible organic and inorganic content of the coprolites. While this study focused on the morphological analysis and paleoecological significance of the coprolites, future studies could consider employing multiple approaches to further investigate the detailed composition and contents of the coprolites.

## STAR★Methods

### Key resources table

**Table:** 

REAGENT or RESOURCE	SOURCE	IDENTIFIER
**Biological samples**		

Fossil coprolite specimens	Institute of Vertebrate Paleontology and Paleoanthropology (IVPP), Chinese Academy of Sciences	IVPP V 27941.1 to IVPP V 27941.55

**Chemicals, peptides, and recombinant proteins**		

Epoxy clear resin	EXAKT Technologies, Inc.	Technovit 7200 VLC

**Deposited data**		

Measurement of sampled specimens	This study	N/A

**Software and algorithms**		

PAST version 4.1	PAST version 4.1^119^	NA
Post-processing of images was performed with Adobe Photoshop CS6 and Adobe Illustrator CS6	Adobe	https://www.adobe.com
Dragonfly Pro Workstation (Version 2022.1 Build 1259)	Object Research Systems, Inc, 2021, Montreal, Canada	https://www.theobjects.com/company/products.html

### Resource availability

#### Lead contact

Further information and requests for resources and materials should be directed to and will be fulfilled by the lead contact, Paul Rummy (paulrummy@ivpp.ac.cn).

#### Materials availability

The 55 coprolites specimens and the thin section samples produced from them are accessioned and accessible at Institute of Vertebrate Paleontology and Paleoanthropology (IVPP), Chinese Academy of Sciences in Beijing, China. Palynology samples are stored in the Paleoecology Laboratory of XTBG in Xishuangbanna, China.

### Experimental model and subject details

We do not have experimental model and subject.

### Method details

#### Fossil material

In the summer of 2018, a field team from the Chinese Academy of Sciences (CAS) and the Vietnam Academy of Science and Technology (VAST) collected over 100 coprolites and coprolite fragments from the Na Duong coal mine in Lang Son Province, Northern Vietnam (Figure 1). Each specimen is labeled with the acronym IVPP V 27941 followed by their respective number.

The studied materials were obtained from the lower units of the Eocene-Oligocene Na Duong Formation in Vietnam. These units primarily consist of sedimentary deposits formed through fluvial and peat-forming processes, including sandstones, siltstones, mudstones with small carbonate concretions, and claystone layers containing lignite-bearing units. The age assignments for the Na Duong fauna remain a topic of debate, with recent studies suggesting much younger age of approximately ∼33Ma (for geological reviews on Na Duong Formation, refer to ref.^115^^,^^118^^,^^120^^,^^121^).

In this study, the term “maximum width” refers to the widest diameter of the coprolite, while the “second width” is measured at a 90-degree angle to the maximum width^28^^,^^83^ (see also Figure S1). The terms “cylindrical”, “oval” and “elliptoidal” are commonly used in literature to describe the morphology of carnivore.^49^^,^^55^^,^^57^ “Longitudinal striations” refers to surface structures originating from muscular pressure during the extrusion of feces through the anal or cloacal sphincter.^57^ The term “cross section” denotes a lateral cut through a coprolite.^55^

#### Neocoprological study

To compare the morphology of the coprolites, descriptions and measurements of extant crocodile feces were derived from Milàn.^55^ Additionally, field visits were made to the Charoen Pokphand (CP) Group Crocodile Farm in Beijing, China, and the Beijing Zoo to collect fresh samples of crocodilians feces (see also Figure S3) and observe the preferable locations (water bodies or terrestrial) for crocodilians to defecate. Observation were made with the naked eyes, and the data were recorded in a notebook.

#### Sampling and thin-sectioning

For microstructural analysis and documentation of any potential bone or soft tissue fragments, three coprolite samples (IVPP V 27941/3, IVPP V 27941/45 and IVPP V 27941/49) were sliced in the mid-section to obtain histological thin sections. For comparison, thin sections were also prepared from the coprolite of an adult extant crocodilian (*Crocodylus siamensis*) from the breeding farm. The specimen were prepared as thin sections following the methodology proposed by Chinsamy and Raath.^122^ Prior to sampling, all specimens underwent CT scans for future 3D printing and restoration purposes. The samples were then embedded in epoxy clear resin Technovit 7200 VLC, vacuumed for a total of 4 hours to remove bubbles, and cured for 12 hours using the EXAKT 22851 machine. Cubical blocks were created and sliced to approximately 250 μm by using a diamond circular saw that was fitted with a diamond-tipped watering blade on the EXAKT 300CP machine. Each section was further polished using a wheel polisher (EXAKT 400CS), starting with 500-grit paper and gradually progressing to finer papers of 1200 and 4000 grit. The final slides had a thickness of approximately 30–40 μm and were polished. The slides were cleaned using a water-filled ultrasonic cleaner to remove microscopic grit before being covered with a glass coverslip. Thin sections were observed and analyzed using normal and polarised light with the lambda compensator on a Zeiss PrimoTech microscope. All laboratory work and observation were conducted in the histology laboratory of IVPP, CAS.

#### Imaging and coloration analysis

Thin sections were examined and described under normal and polarized light with a lambda compensator using a Zeiss PrimoTech microscope. Images were captured using an integrated microscope camera at 200x magnification. Photographs and EDS graphs were compiled using Adobe Photoshop CS6 and Adobe Illustrator CS6. Coloration was determined using the Munsell soil color chart.^123^

#### SEM-EDS analysis

Scanning electron microscopy (SEM) using a Zeiss EVO 25 instrument coupled with energy-dispersive X-ray spectroscopy (EDS) (Oxford X-act) was employed to analyze the coprolite samples. Tiny pieces of the coprolite samples were broken off for analysis, with one sample taken from the surface and another from the inner portion of each coprolite. Three coprolites (IVPP V 27941/3, IVPP V 27941/45, and IVPP 27941/49) were selected for these studies. Additionally, a sediment sample from the Na Duong coal mine was used as a comparison. Prior to analysis, all samples were attached to a stub and coated with a thin layer of gold.

#### Three-dimensional modeling

Non-destructive examination of the coprolite content and generation of 3D models (STL files) were accomplished using Computed Tomography (CT) scanning and a portable 3D scanning device. Specimen IVPP V 27941/3, IVPP V 27941/36, IVPP V 27941/40, IVPP V 27941/46, IVPP V 27941/47, IVPP V 27941/49, IVPP V 27941/51, and IVPP V 27941/53 were scanned using a 240 kV micro-computerized tomography (developed by GE, Model – phoenix v|tome|x m) at the Key Laboratory of Vertebrate Evolution and Human Origins, IVPP, CAS. The scans were performed with a beam energy of 200 kV and a flux of 120 μA, at a resolution of 135.558 μm per pixel. A 360° rotation with a step size of 0.0° was used, resulting in total of 720 projections were reconstructed in a 2048∗2048 matrix of 2000 slices using a two-dimensional reconstruction software developed by the Institute of High Energy Physics, CAS.^124^ The data were output in the TIFF file format and imported into Dragonfly Pro Workstation (Version 2022.1 Build 1259) (Object Research Systems, Inc, 2021, Montreal, Canada).

#### Methods used for palynology

Palynological analysis was conducted to extract common palynomorphs from coprolites and the surrounding matrix obtained from the Na Duong coal mine. Samples were sent to the palynology lab at the Xishuangbanna Tropical Botanical Garden (XTBG), CAS for processing. The samples underwent a series of treatments, including the use of a 10% HCl solution to eliminate carbonates, a 40 % HF solution to remove silicates, and a subsequent treatment with a 10% NaOH solution to remove organic matter. Afterward, an 8-μm mesh was utilized to remove the fine fraction and concentrate pollen through ultrasonic sieving. The identifications of palynomorph types was based on pollen and spores^125^ atlases, as well as modern reference slides from the collection of XTBG, CAS. The resulting macerates were preserved in glycerin in test tubes. The examined samples are currently stored in the Paleoecology Laboratory of XTBG in Xishuangbanna, China. A total of 500 terrestrial pollen grains were counted in each sample, and the percentages of pollen and spores were calculated based on the sum of terrestrial pollen.

#### Life reconstruction

Reconstruction drawings were created with the assistant of Mr. Chung-Tat Cheung under the direction of K.H. and P.R. The paleoenvironment reconstruction was based on various literature sources published on Na Duong fauna. The floral components in the image were actual reconstructions derived from palynology analyses. The figures were prepared using Adobe Photoshop CC 2018 and Adobe Illustrator CC 2018 software. Graphical abstract of the article was contributed by Novia Shin. Additionally, line drawings were made to scale by referring to the original specimens. All measurements of the histological specimens were taken using the scales in the microscope.

#### Ethics

This research is part of a collaborative project between IVPP, CAS, and VAST. The study strictly adhered to all existing regulations, and no permits were required in this context. The collection material described in this study was conducted lawfully and in accordance with the guidelines of the Society of Vertebrate Paleontology. The authors are aware and against any unethical practices of parachute science in paleontology and comes from diverse ethnic and cultural backgrounds. No animals were harmed during the course of this research.

#### Nomenclatural acts

This published work and its associated nomenclatural acts have been registered in ZooBank, an online registration system for the International Code of Zoological Nomenclature (IZCN). The LSID (Life science identifiers) for this publication is urn:lsid:zoobank.org:act:11743765-F8EA-419F-98DA-03B5DE7D9958. The associated information can be accessed through any standard web browser by appending the LSID to the prefix “http://zoobank.org/”.

### Quantification and statistical analysis

For this study, 55 well-preserved specimens were selected for quantitative analyses, excluding the details of IVPP V 27941/35, which can be found in ref.^29^ Measurements of the coprolites were recorded to the nearest millimeter using a Vernier caliper. To preform quantitative paleontological studies, we used PAST version 4.1^119^ software to construct scatter and box-plot diagrams based on the measured coprolite data (see Table S2 for details).

### Additional resources

There is no additional resource to report besides that in the above resource availability section.

## Data Availability

• Data have been deposited at text and supplemental information. • This paper does not report original code. • Any additional information required to reanalyze the data reported in this paper is available from the lead contact upon request.
