# Supplementary material for: Exceptionally well-preserved crocodilian coprolites from the Late Eocene of Northern Vietnam: Ichnology and paleoecological significance
Source: iScience. 2023 Aug 11;26(9):107607. doi: 10.1016/j.isci.2023.107607 (PMC10470398; doi:10.1016/j.isci.2023.107607)
Supplement: Document S1. Figures S1–S3 and Tables S1–S8 [file mmc1.pdf]

iScience, Volume *26*

Supplemental information

Exceptionally well-preserved crocodilian coprolites from the Late Eocene of Northern Vietnam: Ichnology and paleoecological significance

Kazım Halaçlar, Paul Rummy, Jia Liu, Adrian P. Hunt, Truong Van Do, Nguyen Trung Minh, and Tao Deng

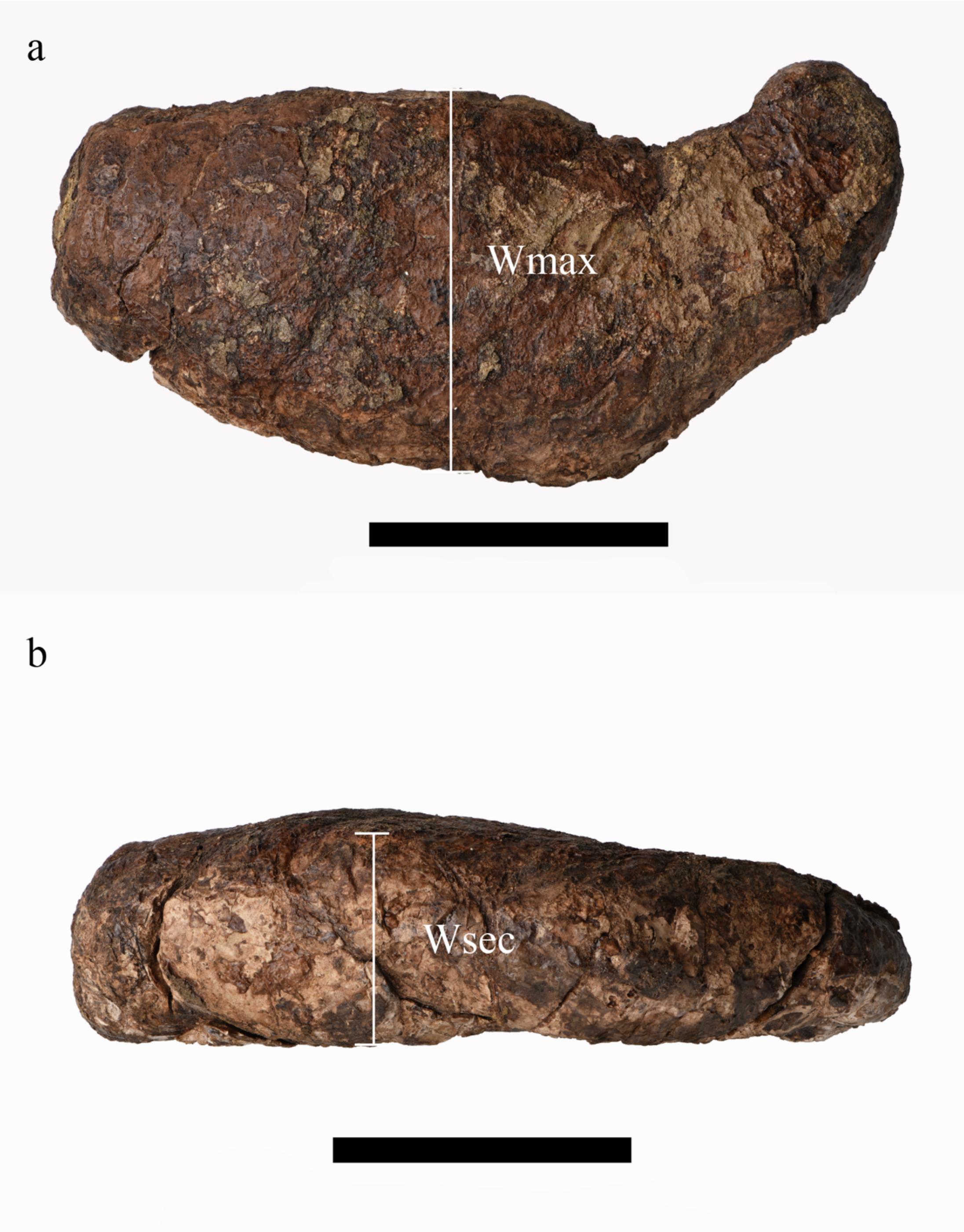

Figure S1. Two width measurement methods were used. Related to STAR Methods. Scale bar equals 5 cm.

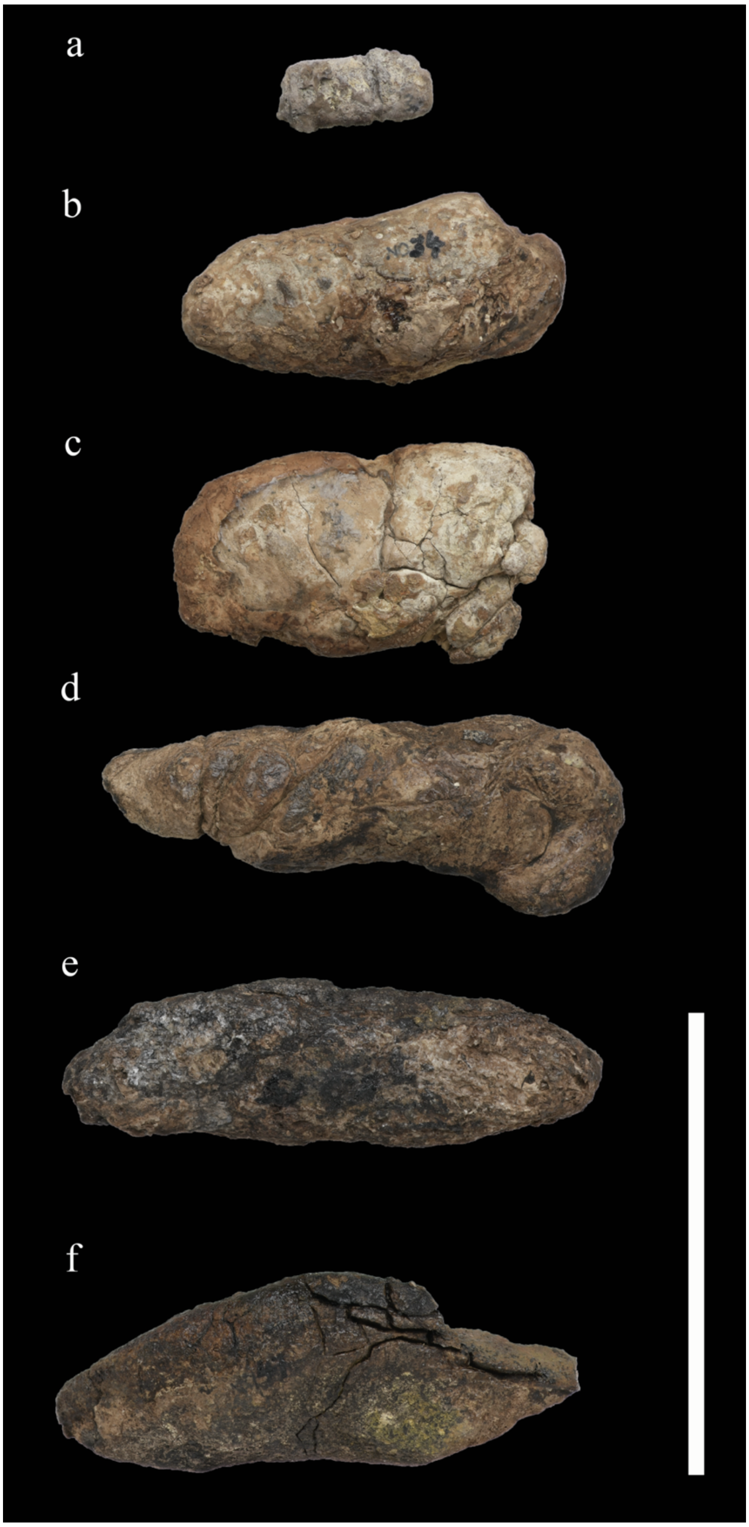

Figure S2. Morphotype A *Crococopros naduongensis* igen. et isp. nov. (Plate 4-Dorsal). Related to Figure 2. Scale bar equals 10 cm.

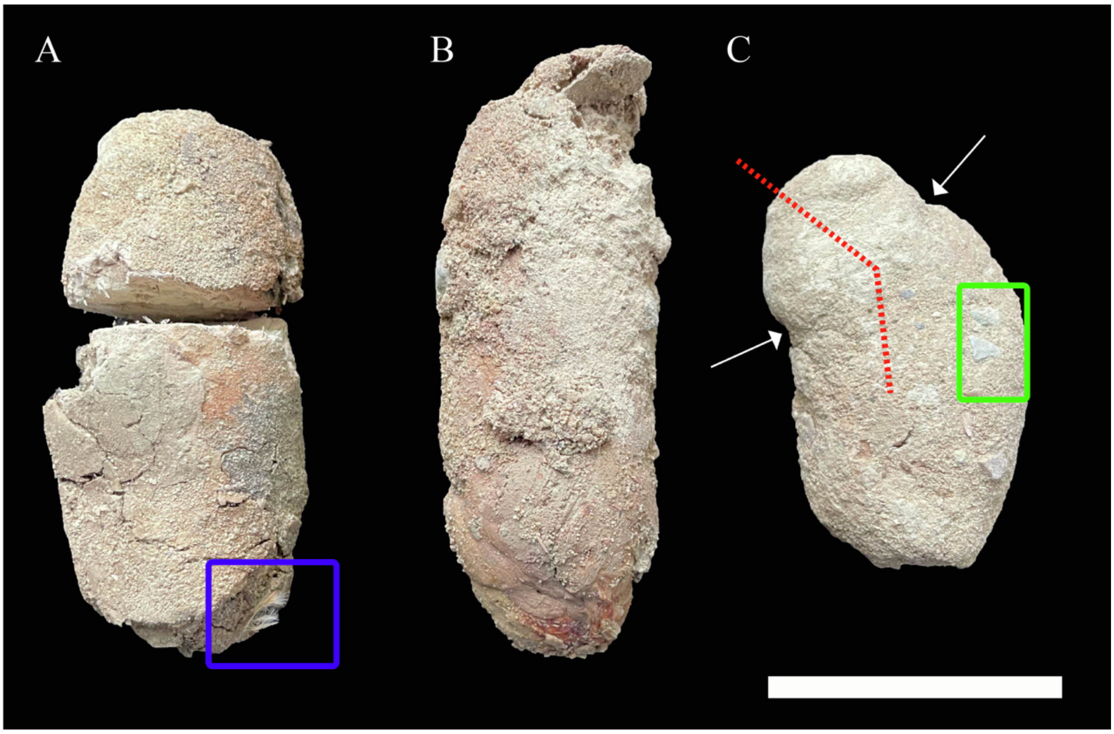

Figure S3. Extant *Crocodylus siamensis* feces from Charoen Pokphand (CP) Group Crocodile Farm. Related to STAR Methods. a.) blue box indicate traces of feathers. b.) sand particles adhere to the fresh feces; and c, green box indicate pebbles which could represent gastroliths, red broken line and arrows show circumferential constriction mark. Scale bar equals to 5 cm.

Table S1. Past studies on purported/possible crocodilian coprolites in history (modified from Hunt and Lucas, 2010[1]). Related to Figure 5.

Identification of Coprolite Producer	Age	Location	References	Notes
Possibly crocodilian	Early Cretaceous	Belgium	Bertrand (1903)[2]; Abel (1935)[3]	
Crocodylomorphs	Early Cretaceous	Tunisia	Dridi (2022)[4]	
Crocodilian	Early Cretaceous	France	Rozada et al. (2020)[5]	
Crocodylomorph	Late Cretaceous	Brazil	Souto (2010)[6]	
Crocodilian	Late Cretaceous	Canada	Waldman (1970)[7]	Fisher (1981)[8] doubts crocodilian origin
Mariliasuchus amarali	Late Cretaceous	Brazil	Nobre et al. (2008)[9]	
Deinosuchus rugosus, *Borealosuchus*	Late Cretaceous	USA	Schwimmer (2002)[10]; Harrell and Schwimmer (2010)[11]	Hunt and Lucas (2010)[1] consider the putative *Deinosuchus* coprolites to be concretions
Crocodylomorphs	Late Cretaceous	Brazil	De Oliveira et al. (2021)[12]	
Pseudosuchia	Late Triassic	Greenland	Milàn et al. (2021)[13]	
Leidyosuchus formidabilis	Paleocene	USA	Sawyer (1981)[14]	
Asiatosuchus nanlingensis	Paleocene	China	Young (1964)[15]	
Eoalligator chuyii	Paleocene	China	Young (1964)[15]	
Crocodilian	Paleocene	USA	Sawyer (1988)[16]	
Crocodile	Paleocene	Denmark	Milàn (2010)[17]	
Crocodilian	Upper Paleocene	USA	Godfrey et al. (2020)[18]	
Crocodilian	Eocene	Argentina	Krause and Piña (2012)[19]	
Crocodile gastrointestinal contents or coprolites	Eocene	Germany	Walther and Weigelt (1932)[20]; Fikentscher (1933)[21]; Numberger (1933, 1934)[22]; Voigt (1934)[24]	
Crocodilian	Eocene	USA	Jepsen (1963)[25]	Fisher (1981)[8] doubts crocodilian origin
Crocodilian (?)	Eocene	France	Robert (1832-1833)[26]	
Crocodilian	Early Eocene	USA	Hunt et al. (2012)[27]	
Crocodilian	Oligocene	Denmark	Milàn et al. (2018)[28]	
Possibly crocodile or large fish	Miocene	USA	Wetmore (1943)[29]; Davis and Briggs (1995)[30]	Contains feather. Fisher (1981)[8] doubts crocodilian origin and suggests it may not be crocodile at all
Crocodilian	Miocene	USA	Stirton (1959)[31]	
Crocodilian	Miocene	Czech Republic	Mikulas and Dvorak (2010)[32]	
Crocodilian	Late Miocene	Venezuela	Dentzien-Dias et al. (2017)[33]	
Crocodilian	Plio-Pleistocene	Ethiopia	Butzer (1971)[34]	
Crocodylus niloticus	Mid Holocene	Sahara region	De Smet (1998)[35]	

Table S2. List of all the well-preserved coprolites collected from the Na Duong Formation in Northern Vietnam. Related to STAR Methods.

Specimen Number (IVPP V 27941/)	Length	WMAX	Wsec	Variation
**1**	102.15	44.11	18	A
**2**	79.82	32.7	19.79	A
**3**	103.22	38.68	19.4	A
**4**	79.75	36.73	20.1	A
**5**	109.33	47.14	27.54	A
**6**	59.4	31.35	21.78	A
**7**	111.67	45.05	27.41	A
**8**	65.95	29.37	15.57	A
**9**	97.82	37.08	18.29	A
**10**	80.98	33.81	20.09	A
**11**	88.79	38.03	26.53	A
**12**	74.75	33.4	18.03	A
**13**	81.1	39.61	19.35	A
**14**	80.72	32.29	26.77	A
**15**	92.76	36.53	22.61	A
**16**	62.94	35.2	23.27	A
**17**	72.28	34.6	22.94	A
**18**	78.93	46.01	28.58	A
**19**	92.29	30.64	22.14	A
**20**	89.87	29.88	25.29	A
**21**	105.21	46.61	38.41	A
**22**	99.76	54.51	35.02	A
**23**	102.56	51.88	35.22	A
**24**	72.1	38.5	25.65	A
**25**	104.31	40.22	31.82	A
**26**	84.31	40.58	30.36	A
**27**	145.24	34.6	23.41	A
**28**	46.24	34.6	23.41	A
**29**	33.38	15.17	11.1	A
**30**	83.02	37.77	33.23	A
**31**	79.79	45.28	22.71	A
**32**	111.05	40.58	18.84	A

**33**	114.33	35.73	23.76	A
**34**	113.95	44.05	23.78	A
**35**	106	47	21	A
**36**	98.39	44.45	40.52	B
**37**	82.67	49.83	36.26	B
**38**	109.5	45.51	34.58	B
**39**	94.43	44.68	44.12	C
**40**	77.93	47.36	34.64	C
**41**	97.32	51.55	42.16	C
**42**	57.82	42.4	33.73	C
**43**	92.51	47.16	41.43	C
**44**	120.49	63.46	45.08	D
**45**	116.81	64.83	43.03	D
**46 (Holotype)**	144.29	64.28	35.54	D
**47**	201.69	58.33	41.7	D
**48**	63.58	30.39	20.43	U
**49**	121.28	26.83	25.24	U
**50**	68.48	41.06	30.28	U
**51**	65.74	44.72	17.98	U
**52**	72.86	41.16	37.77	U
**53**	69.81	52.02	24.31	U
**54**	80.42	58.34	27.28	U
**55**	77.51	60.69	24.92	U

Table S3. Ichno-morphotypes of *Crococopros naduongensis* igen. et isp. nov. as to their morphologies. Related to Figure 2 and 3.

	NS	Longitudinal View	Transversed View
MorA	25	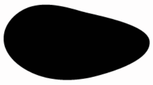	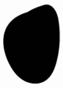
MorB	4	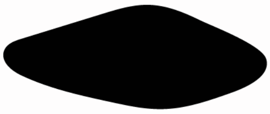	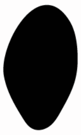
MorC	14	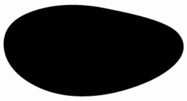	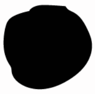
MorD	4	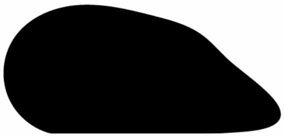	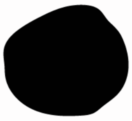

Table S4. Possible matching between morphotypes of *Crococopros naduongensis* igen. et isp. nov. and types of Na Duong crocodilians. Related to Figure 4. Abbreviation: BL – Body length; ND – Na Duong coal mine.

Type	Feature	Morphotype
Brevirostrine 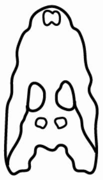	2/3 all ND croc findings<br>BL - 2 meters	A and B
Longirostrine Crocodilian 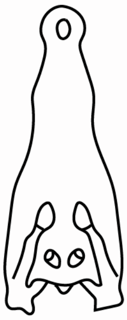	1/3 all ND croc findings<br>BL - 6 meters	C and D
Longirostrine Gavialoid 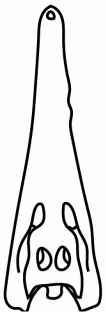	One specimen finding<br>BL - 6 meters	None

Table S5. Morphotypes of *Crococopros naduongensis* igen. et isp. nov. coprolites. Related to STAR Methods.

Morphotype	Number of Specimen	Average Length	Max length	Min length	Average Wmax	Max Wmax	Min Wmac	Average Wsec	Max Wsec	Min Wsec
	55	98	201	33	47.6	64	15	33.4	45	11
MorA	35	88.7	145	33	38.2	54.5	15	24	38.5	11
MorB	3	96	109.5	82.6	46.5	49.8	44.4	37	40.5	34.5
MorC	5	84	97	57.8	46.6	51.5	42.4	39	44	33.7
MorD	4	145	201	116	62.7	64	58	41	45	35.5
Uncategorized	8	77	121	63	44	60	26	26	37	18

Table S6. SEM images and EDS analyses of Na Duong coal mine sediment and *Crococopros naduongensis* igen. et isp. nov. specimens. Related to STAR Methods. Abbreviations as indicated in the text.

Sample number	Element compositions	SEM images	Elemental graphs
Na Duong sediment	C, K, O, Fe, Na, Al, Si, S, K	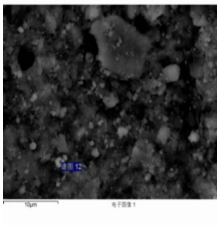	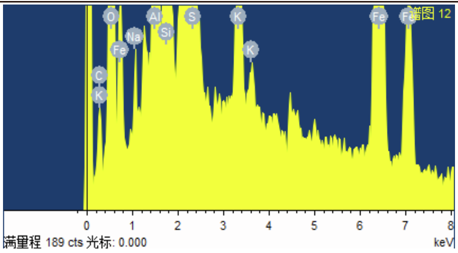
IVPP V 27941/3	C, Ca, P	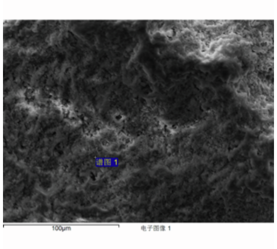	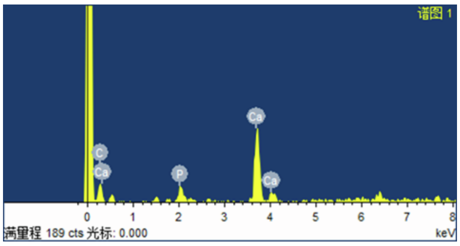
IVPP V 27941/45	C, Ca, P, Fe	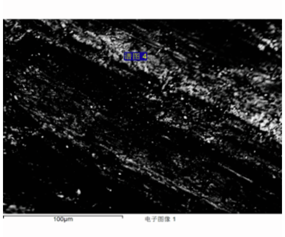	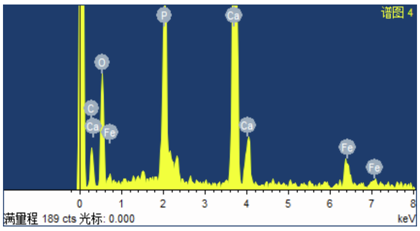
IVPP V 27941/49	C, Ca, P, Fe	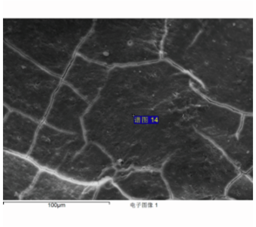	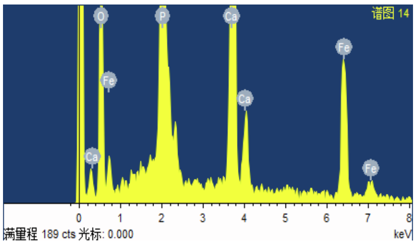

Table S7. A list of common palynomorphs retrieved from palynology analysis on *Crococopros naduongensis* igen. et isp. nov. specimens and their surrounding rocks in Na Duong coal mine. Related to STAR Methods.

	**Palynomorph types**
**Gymnosperm**	*Abietineaepollenites* sp., *Ephedripites* sp., *Pinuspollenites* sp., *Podocarpidites* sp., *Taxodiaceaepollenites hiatus*
**Angiosperm**	*Aceripollenites* sp., *Alangiopollis* sp., *Alnipollenites* sp., *Alnipollenites verus*, Arecaceae-type, *Betulaepollenites plicoides*, *Betulaepollenites* sp., *Boehlensipollis* sp., *Carpinipites* sp., *Caryapollenites granulatus*, *Caryapollenites polarannulus*, *Celtispollenites* sp., *Coriaripites minor*, *Cornaceoipollenites* sp., *Cupanieidites* sp., *Cupuliferoipollenites oviformis*, *Cupuliferoipollenites pusillus*, *Cyrillaceaepollenites* sp., *Engelhardtioidites* sp., *Euphorbiacites* sp., *Faguspollenites* sp., *Ficus*-type, *Fraxinoipollenites* sp., *Fupingopollenites imbecillus*, *Fupingopollenites* sp., *Haloragacidites* sp., *Ilexpollenites* sp., *Juglanspollenites* sp., *Lemna*-type, *Liquidambarpollenites* sp., *Magnolipollis* sp., *Meliaceoidites* sp., *Momipites* sp., *Monocolpopollenites* sp., *Moraceoipollenites* sp., *Myricipites* sp., *Nothopollenites* sp., *Nymphaeacidites* sp., *Oleoidearumpollenites* sp., *Operculumpollis operculatus*, *Ostryoipollenites* sp., *Persicarioipollis communis*, *Persicarioipollis welzowensis*, *Platycaryapollenites* sp., *Polycolpites* sp., *Potamogetonacidites* sp., *Pterocaryapollenites* sp., *Quercoidites* sp., *Ranunculacidites* sp., *Retitricolpites alveolatus*, *Retitricolpites* sp., *Rhoipites* sp., *Rutaceoipollenites* sp., *Rutaceoipollis* sp., *Salixipollenites* sp., *Sapotaceoidaepollenites* sp., *Shorea*-type, *Sparganiaceaepollenits sparganioides*, *Striacolporites* sp., *Tricolpites* sp., *Tricolpopollenites megagranulatus*, *Tricolpopollenites psilatus*, *Tricolpopollenites* sp., *Ulmipollenites* sp., *Ulmoideipites* sp., *Zelkovaepollenites* sp.
**Fern**	*Deltoidospora* sp., *Osmundacidites microquintus*, *Polypodiaceaesporites* sp.
**Algae**	*Pediastrum* sp. (aff. *Pediastrum simplex* var. *sturmii*), *Multispinula* sp.

Table S8. Paleogeography comparisons between Eocene localities of crocodilian coprolites. Related to Figure 5.

Locality\Factors	Age	Coprolite morphology	Coprolite Inclusion	Coprolite geochemical	Coprolite Palynology	Coprolite Cysts	Climate	Environment	Paleoecology	Fauna	Flora	References
**Na Duong**	Late Eocene early Oligocene	circular cross section, tapered and flattened ends, CCM	no inclusion	P, C, Ca	5 of gymnosperms, 66 of angiosperms, 3 of ferns, and 2 of algae	no	tropical/ subtropical paleoclimates	a freshwater basin with standing or slowly flowing water, under humid climatic conditions, without any dry condition	wet forest swamp combining the swamp, lake-margin, and lacustrine facies association	6 taxa turtles, 3 crocs morphotypes	Megaflora, deciduous oak and shorea	This work. 36. Böhme et al. (2013)[36], Wysocka et al. (2020)[37]
**Viborg Formation**	Late Eocene early Oligocene	tapering towards the ends, circular CS, CCM150	no inclusion	NA	NA	dinoflagellate cysts	warm-temperate to subtropical.	The Viborg Formation was deposited in a relatively deep marine environment (> 300 m of water) at a minimum of 150 km from the shoreline.	Spit systems and barrier–lagoon complexes formed in between the major delta lobes. The coastline was dominated by mangrove swamp, and on elevated areas in the hinterland sequoia and oaks vegetated the landscape.	Cetacea and sharks	no	Milan et al. (2018)[28]
**Zaysan Basin**	late Eocene	rounded ends and cylindrical CS	no inclusion	NA	NA	NA	warm-temperate to subtropical ?	Lacustrine and/or lacustrine margin deposits	old shoreline or beach of paleo-lake	ostracods, unionid bivalves, non-marine gastropods, freshwater fishes, turtles, mammals, and Crocodilian.	megaflora, charophytes,	Lucas et al. (2012)[38]
**Nanxiong Localities (L4 L5)**	Late Eocene	Slightly flatted and curved with more or less distinct tapered ends	no inclusion	P2O5 (%11.35)	NA	NA	?	?	?	2 types of crocs	?	Young (1964)[15]
**Pico Salamanca (Las flores formation)**	Late Paleocene early Eocene	fusiform and six have a subcylindrical shape. Transverse sections show sub-circular to sub-elliptical shapes	no inclusion	O, Ca, P, C, Si	NA	NA	tropical/ subtropical paleoclimates	The sections were deposited within a fluvial mixed-load system	alluvial plain with meandering channels and broad floodplains	no crocs but central Patagonia has two types	non	Krause and Piña (2012)[19]

References

1. Hunt, A., and Lucas, S. (2010). Crocodylian coprolites and the identification of the producers of coprolites. New Mexico Museum of Natural History and Science Bulletin *51*, 219–226.
2. Bertrand, C.E. (1903). Les coprolithes de Bernissart. I. partie: Les coprolithes qui ont ete attribues aux Iguanodons. Mémoire du Musée Royal d'Histoire Naturelle de Belgique, Brussels *1*, 1–154.
3. Abel, O. (1935). Vorzeitliche Lebensspuren. Jena, G. Fischer, pp. 644.
4. Dridi, J. (2022). First report of diverse vertebrate coprofaunas from the Lower Cretaceous of Tunisia. Cretaceous Research 105192. 10.1016/j.cretres.2022.105192.
5. Rozada, L., Allain, R., Vullo, R., Goedert, J., Augier, D., Jean, A., Marchal, J., Peyre de Fabrègues, C., Qvarnström, M., and Royo-Torres, R. (2021). A Lower Cretaceous Lagerstätte from France: a taphonomic overview of the Angeac-Charente vertebrate assemblage. Lethaia *54*, 10.1111/let.12394.
6. Souto, P.R.F. (2010). The crocodylomorph coprolites from Bauru Basin, Upper Cretaceous, Brazil. New Mexico Museum of Natural History and Science Bulletin *51*, 219–226.
7. Waldman, M., Hopkins, W.S. (1970). Coprolites from the Upper Cretaceous of Alberta, Canada, with a description of their microflora. Canadian Journal of Earth Sciences *7*, 1295e1303.
8. Fisher, D.F. (1981) Crocodilian scatology, microvertebrate concentrations, and enamel-less teeth. Paleobiology *7*, 262–275.
9. Nobre, P.H., de Souza Carvalho, I., de Vasconcellos, F.M., and Souto, P.R. (2008). Feeding behavior of the Gondwanic Crocodylomorpha *Mariliasuchus amarali* from the Upper Cretaceous Bauru Basin, Brazil. Gondwana Research *13*, 139–145.
10. Schwimmer, D.R. (2002). King of the crocodylians: The paleobiology of *Deinosuchus*. Bloomington, (Indiana University Press), pp. 221.
11. Harrell, S.D., and Schwimmer, D.R. (2010). Coprolites of *Deinosuchus* and other crocodylians from the Upper Cretaceous of western Georgia, USA. New Mexico Museum of Natural History and Science Bulletin *51*.
12. De Oliveira, F., Santucci, R., Oliveira, C., and de Andrade, M.B. (2021). Morphological and compositional analyses of coprolites from the Upper Cretaceous Bauru Group reveal dietary habits of notosuchian fauna. Lethaia 10.1111/let.12431.
13. Milàn, J., Sanei, H., Clemmensen, L., Mau, M., Mateus, O., and Rudra, A. (2021). A possible phytosaurian (Archosauria, Pseudosuchia) coprolite from the Late Triassic Fleming Fjord Group of Jameson Land, central East Greenland. Bulletin of the Geological Society of Denmark *69*, 71–80. 10.37570/bgsd-2021-69-05.
14. Sawyer, G.T. (1981). A study of crocodilian coprolites from Dannagan Creek quarry (Paleocene-North Dakota). Scientific Publications of the Science Museum of Minnesota new series *5*, 3–29.
15. Young, C.C. (1964). New fossil crocodiles from China. Vertebrata PalAsiatica *8*, 189–208.
16. Sawyer, G.T. (1988). Coprolites of the Black Mingo Group (Paleocene) of South Carolina. Transactions of the American Philosophical Society, New Series *88*, 221–228.
17. Milàn, J. (2010). Coprolites from the Danian limestone (Lower Paleocene) of Faxe Quarry, Denmark. New Mexico Museum of Natural History and Science Bulletin *51*, 215–218.
18. Godfrey, S., Alford, A., Collareta, A, and Weems, R. (2020). A Paleocene vertebrate-bitten crocodilian coprolite from Liverpool Point. Neues Jahrbuch für Geologie und Paläontologie - Abhandlungen *296*, 237–244. 10.1127/njgpa/2020/0904.
19. Krause, J., and Piña, C. (2012). Reptilian Coprolites in the Eocene of Central Patagonia, Argentina. Journal of Paleontology *86*, 527–538. 10.2307/41480215.
20. Walther, J. and Weigelt, J. (1932). Die Eozäne Lebewelt in der Braunkohle des Geiseltals. Nova Acta Leopoldiana N. F. *1*, p. 1–27.
21. Fikentscher, R. (1933). Koproporphyrin in Tertiären Krokodilkot. Zoologischer Anzeiger *103*, 289–295.
22. Nürnberger, L. (1933). Koproporphyrin in Tertiären Krokodilkot. Nova Acta Leopoldina. Halle N Ser. *1*, 324–325.
23. Nürnberger, L. (1934). Koproporphyrin in Tertiären Krokodilkot. Forschungen und Fortschriften. *9*, 39–40.
24. Voigt, E. (1934). Die Fische aus der mitteleozänen Braunkohle des Geiseltales,mit besonderer Berüchsichtigung der erhaltenen Weichteile. Nova Acta Leopoldiana N. F. *2*, 21–146.

25. Jepsen, G.L. (1963). Eocene vertebrates, coprolites and plants in the Golden Valley Formation in western North Dakota. Geological Society of America Bulletin *74*, 673–684.
26. Robert, E. (1832–1833). Sur les coprolithes trouvés à Passy. Bulletin Societe Géologique de France Série 1 *3*, 72–73.
27. Hunt, A., Lucas, S., Spielmann, J., Cantrell, A. and Suazo, T. (2012). A. New marine coprofauna from the Beeman formation (Late Pennsylvanian: Late Missourian), Sacramento Mountains, New Mexico, USA. New Mexico Museum of Natural History and Science Bulletin *57*, 193–196.
28. Milàn, J., Rasmussen, E., and Dybkjaer, K. (2018). A crocodilian coprolite from the lower Oligocene Viborg Formation of Sofienlund Lergrav, Denmark. Bulletin of the Geological Society of Denmark *66*, 181–187.
29. Wetmore, A. (1943). The occurrence of feather impressions in the Miocene deposits of Maryland. Auk *60*, 440–441.
30. Davis, P.G., and Briggs, D.E.G. (1995). Fossilization of feathers. Geology *23*, 783–786.
31. Stirton, R.A. (1959). Time, life and man. New York, John Wiley 558 p.
32. Mikuláš, R., and Dvorák, Z. (2010). Possible crocodylian bite traces, Miocene of the Most Basin (Czech Republic). New Mexico Museum of Natural History and Science Bulletin *51*, 191–194.
33. Dentzien-Dias, P., Hunt, A.P., Lucas, S.G., Francischini, H., and Gulotta, M. (2020). Coprolites from shallow marine deposits of the Nanjemoy Formation, Lower Eocene of Virginia, USA. Lethaia https://doi.org/10.1111/let.12380.
34. Butzer, K.W. (1971). Lower Omo Basin: Geology, fauna and hominids of Plio-Pleistocene formations. Naturwissenschaften *58*, 7–16.
35. De Smet, K. (1998). Status of the Nile Crocodile in the Sahara Desert. Hydrobiologia *391*, 81–86.
36. Böhme, M., Aiglstorfer, M., Antoine, P.O., Appel, E., Métais, G., Phuc, L., Schneider, S., Setzer, F., Tappert, R., Tan, D., and Prieto, J. (2013). Na Duong (northern Vietnam) – an exceptional window into Eocene ecosystems from Southeast Asia. Zitteliana A *53*, 120–167.
37. Wysocka, A., Phan, P., Ewa, D., Urszula, C., Do, T., Anna, F., Nguyen, C., Dang, T., Nguyen, H., Hoang, T., and Radosław, S. (2020). The Na Duong Basin (North Vietnam): A key for understanding Paleogene basin evolution in relation to the left-lateral Cao Bang-Tien Yen Fault. Journal of Asian Earth Sciences *195*, 104350. 10.1016/j.jseaes.2020.104350.
38. Lucas, S., Spielmann, J., Hunt, A.P., and Emry, R. (2012). Crocodylian coprolites from the Eocene of the Zaysan Basin, Kazakstan. New Mexico Museum of Natural History and Science Bulletin *57*, 319–324.
